# Transcriptome-wide characterization of bHLH transcription factor genes in *Lycoris radiata* and functional analysis of their response to MeJA

**DOI:** 10.3389/fpls.2022.975530

**Published:** 2023-01-10

**Authors:** Ning Wang, Xiaochun Shu, Fengjiao Zhang, Zhong Wang

**Affiliations:** Jiangsu Key Laboratory for the Research and Utilization of Plant Resources, Institute of Botany, Jiangsu Province and Chinese Academy of Sciences (Nanjing Botanical Garden Mem. Sun Yat-Sen), Nanjing, China

**Keywords:** *Lycoris radiata*, bHLH transcription factors, expression patterns, MeJA treatment, regulatory networks

## Abstract

As one of the biggest plant specific transcription factor (TF) families, basic helix–loop–helix (bHLH) protein, plays significant roles in plant growth, development, and abiotic stress responses. However, there has been minimal research about the effects of methyl jasmonate (MeJA) treatment on the *bHLH* gene family in *Lycoris radiata* (L’Her.) Herb. In this study, based on transcriptome sequencing data, 50 putative *L. radiata bHLH (LrbHLH)* genes with complete open reading frames (ORFs), which were divided into 20 bHLH subfamilies, were identified. The protein motif analyses showed that a total of 10 conserved motifs were found in LrbHLH proteins and motif 1 and motif 2 were the most highly conserved motifs. Gene ontology (GO) and Kyoto Encyclopedia of Genes and Genomes (KEGG) enrichment analysis of *LrbHLH* genes revealed their involvement in regulation of plant growth, jasmonic acid (JA) mediated signaling pathway, photoperiodism, and flowering. Furthermore, subcellular localization revealed that most LrbHLHs were located in the nucleus. Expression pattern analysis of *LrbHLH* genes in different tissues and at flower developmental stages suggested that their expression differed across lineages and might be important for plant growth and organ development in *Lycoris*. In addition, all *LrbHLH* genes exhibited specific spatial and temporal expression patterns under MeJA treatment. Moreover, protein-protein interaction (PPI) network analysis and yeast two-hybrid assay showed that numerous LrbHLHs could interact with jasmonate ZIM (zinc-finger inflorescence meristem) domain (JAZ) proteins. This research provides a theoretical basis for further investigation of *LrbHLHs* to find their functions and insights for their regulatory mechanisms involved in JA signaling pathway.

## Introduction

*Lycoris* Herbert belongs to the family Amaryllidaceae, consisting of almost 20 species native to eastern Asia moist warm temperate woodlands (Shi et al., 2006). Plants in the genus *Lycoris* have been utilized as traditional medicine preparation, and more than 110 potent structurally distinct Amaryllidaceae alkaloids were isolated or identified for extensive pharmacological and phytochemical investigations (Cahlíková et al., 2020). For example, according to Compendium of Materia Medica, *Lycoris* plants are described as potent antidotes to poison, effective agents to alleviate pain and relieve inflammation, and diuretic drugs (Jin, 2011). Amaryllidaceae alkaloids from *L. radiata* bulbs were traditionally used for treating sore carbuncle, neurodegenerative diseases, poliomyelitis, suppurative wounds and ulcers (Feng et al., 2011; Chen et al., 2016; Shen et al., 2019).

Many genes potentially participated in Amaryllidaceae alkaloid and anthocyanin biosynthesis as well as sucrose degradation have been identified in *Lycoris* plants through transcriptome sequencing (Wang et al., 2017; Park et al., 2019; Yue et al., 2019; Li et al., 2020a; Wang et al., 2021). For example, genes involved in Amaryllidaceae alkaloid biosynthesis including phenylalanine ammonia lyase (PAL, Li et al., 2018), cinnamate 4-hydroxylase (C4H, Li et al., 2018), tyrosine decarboxylase (TYDC, Wang et al., 2019b); norbelladine synthase (NBS, Li et al., 2020a); norbelladine 4’-O-methyltransferase (N4OMT, Li et al., 2019a), and cytochrome P450 monooxygenase 96T1 (CYP96T1, Li et al., 2020a) have been characterized from *Lycoris* plants. Besides, key functional genes involved in anthocyanin metabolism during flower development in *L. radiata* have also been characterized (Wang et al., 2021). Moreover, a relatively active cell wall invertase (CWIN)-catalyzed apoplasmic sucrose cleavage pattern at the competence stage have been found to increase *L. sprengeri* bulblet regeneration (Ren et al., 2021), and the mode of sucrose degradation could be identified as a metabolic marker for early vegetative propagation in bulbs of *Lycoris* (Ren et al., 2022). Considering transcription factors (TF) are important gene expression switches that activate or repress the expression of specific target genes by interacting with *cis*-elements in the gene promoter region, regulating various biological processes including plant development, growth, biosynthesis of secondary metabolites, and response to stresses (Sun et al., 2018), it is necessary to investigate whether and how TFs participate the biological processes in *Lycoris* plants.

The bHLH TF family is the second largest class of TFs widely distributed in plants, animals, and microorganisms (Sun et al., 2018). The bHLH domain comprises approximately 60 amino acids with a basic amino acid region and a helix–loop–helix region (Toledo-Ortiz et al., 2003). The basic region is located on the N-terminal side of the bHLH domain and comprises around 15-20 amino acids (Feller et al., 2011; Sun et al., 2018). It forms a DNA-binding domain, which regulates G-box and E-box DNA binding activity on genes. In contrast, the HLH region contains approximately 40-50 amino acids in the C-terminal domain with two alpha helixes separated by a loop of variable length (Murre et al., 1989). It promotes the interaction with other bHLH proteins and the formation of homodimers and heterodimeric among bHLH TF. The basic region of plant bHLH domains comprises a highly conserved His5-Glu9-Arg13 sequence that binds to the E-box element (5′-CANNTG-3′) (Sun et al., 2018). bHLH proteins have been clarified to 15–26 subfamilies in numerous plants, including rice (Carretero-Paulet et al., 2010), *Arabidopsis* (Carretero-Paulet et al., 2010), Chinese cabbage (Song et al., 2014), tomato (Sun et al., 2015), *Brachypodium distachyon* (Niu et al., 2017), peanut (Gao et al., 2017), cotton (Lu et al., 2018), grape (Wang et al., 2018), maize (Zhang et al., 2018a), and jujube (Li et al., 2019b), *Cucumis sativus* L. (Li et al., 2020b), Capsicum (Liu et al., 2021).

In plants, bHLH TFs influence the development of shoot branches (Komatsu et al., 2001), microspores (Sorensen et al., 2003), fruits and flowers (Gremski et al., 2007), trichomes (Morohashi et al., 2007), stomata (Pillitteri et al., 2007), and roots (Menand et al., 2007). The bHLH TFs are also implicated in various signal transduction and anabolic pathways, including anthocyanin synthesis (Sakamoto et al., 2001), tryptophan production (Smolen et al., 2002), light signal transduction (Castillon et al., 2007), and gibberellin production (Arnaud et al., 2010). For example, the *Arabidopsis* bHLH protein AtPIF3 (PHYTOCHROME INTERACTING FACTOR3) and AtPIF4 (PHYTOCHROME INTERACTING FACTOR4) interact with phytochrome to control the expression of genes regulating light response (Toledo-Ortiz et al., 2003). In flavonoid biosynthesis, bHLH proteins serve as R2R3-MYB and WD40 cofactors in the synthesis of MYB-bHLH-WD40 (MBW) complex (Xie et al., 2012). TRANSPARENT TESTA8 (TT8, AtbHLH42) regulates anthocyanin and procyanidin synthesis in vegetative organs by forming MBW (TT2-TT8-TTG1) complexes (Baudry et al., 2004). The bHLH proteins also affect stress responses, such as drought response (Sun et al., 2018), heat response (Koini et al., 2009), salt response (Verma et al., 2020), and cold response (Feng et al., 2012). The expression level of *AtbHLH92* is upregulated under salt, drought, and cold stresses (Jiang et al., 2009). In rice*, OsbHLH148* is associated with jasmonic acid (JA) signaling and participates in both drought stress and trauma response (Seo et al., 2011). *OsRERJ1* bHLH TF physically associates with *OsMYC2* to participate in the transcriptional induction of JA-mediated stress responsive genes thus defensing against herbivory and bacterial infection (Valea et al., 2021). In apple, *MdbHLH3* expression was induced at low temperature and responsible for anthocyanin accumulation and fruit coloring (Xie et al., 2012).

In this study, 50 *LrbHLH* genes were identified from *L. radiata* transcriptome data (Wang et al., 2021), and their motif pattern and phylogenetic relationship between *Arabidopsis* and *L. radiata* were analyzed. Subcellular localization analysis revealed that LrbHLH proteins were mainly localized in the nucleus. The expression levels of *LrbHLH* genes changed in different tissues and under methyl jasmonate (MeJA) treatment. The probable protein-protein interaction **(**PPI) of LrbHLHs were predicted and confirmed using yeast two-hybrid (Y2H) system. Our results provide important insights into the *bHLH* genes in *L. radiata* and lay a foundation for further investigation of *bHLH* gene function in the biological pathway.

## Materials and methods

### Plant materials and treatments

*Lycoris radiata* (L’Her.) Herb. was planted in Experimental Plantation of the Institute of Botany, Jiangsu Province and Chinese Academy of Sciences, Nanjing, China. The seedlings with the same or similar sizes (2.8– 3.2 cm) in diameter were transferred into plastic pots with a mixture of vermiculite and soil (1:1, v/v) and maintained in a plant growth chamber under the following conditions: 16 h light/8 h dark cycle at 25°C/22°C, and 120 μmol m^-2^ s^-1^ irradiation). After one week of maintenance, the seedlings were subjected to 100 μmol L^−1^ MeJA for 0 h, 6 h, 12 h, 24 h, and 36 h, and roots were sampled for gene expression analysis. Each treatment was replicated three times. Tissue-specific transcription profiles of 50 *LrbHLH* genes were explored in the petals, flower-stalks, gynoeciums, stamens, leaves, seeds, roots, and bulbs of these plants. All samples were immediately frozen in liquid nitrogen and stored at -80°C.

### Transcriptome-wide identification and expression profiling of *LrbHLH* genes

The *L. radiata* transcriptome database during four flower development stage with 87,584 unigenes was used for potential *LrbHLH* searching (Wang et al., 2021). 162 AtbHLH proteins downloaded from TAIR (*Arabidopsis* Information Resource database, https://www.arabidopsis.org/) were utilized to determine sequence homology with *L. radiata* transcripts from the database by basic local alignment (BLASTn). The hidden Markov model (HMM) profile of the bHLH domain (protein family ID: PF00010) obtained from the Pfam protein family database (http://pfam.xfam.org; Mistry et al., 2021) was used to search candidate *LrbHLH* genes. Then, we verified the bHLH domain in the predicted LrbHLH transcription factors utilizing NCBI Batch CD-Search Tool (https://www.ncbi.nlm.nih.gov/Structure/bwrpsb/bwrpsb.cgi) with default parameters. This characteristic was deemed to have a high-confidence association with the conserved domain. Sequences predicted as specific hits were retained for further analysis (**Supplementary Table 1**). Furthermore, PFAM and SMART (http://smart.embl-heidelberg.de/; Letunic et al., 2021) databases were used for verifying the bHLH domain in all candidate protein sequences. Finally, the ExPASy website (https://web.expasy.org/protparam; Artimo et al., 2012) was utilized to determine the full length of amino acid sequences, isoelectric points (PI), molecular sizes (MW), and protein instability index.

### Phylogenetic tree and protein motif analyses of LrbHLH proteins

The phylogenetic tree of bHLHs from *L. radiata* and *A. thaliana* was constructed with MEGA7 using the neighbor-joining method (https://www.megasoftware.net/; Kumar et al., 2016a). LrbHLH proteins were then classified according to their phylogenetic relationships with AtbHLH proteins. The online tool MEME (Multiple EM for Motif Elicitation, version 5.1.1) was utilized to search for the conserved motifs of LrbHLH proteins (https://meme-suite.org/meme/tools/meme; Bailey et al., 2015), with motif number and width of 12-50 for each gene. Motifs were also searched in protein databases using the SMART program (http://smart.embl.de/).

### The GO and KEGG annotation of LrbHLHs

Gene Ontology (GO) functional annotations were conducted using KOBAS (Bu et al., 2021). The top 20 functional terms for credibility in the three categories (biological processes, cellular components, and molecular function) were selected for visualization. KEGG annotations were completed utilizing KASS (Kanehisa et al., 2017).

### Expression profiles analysis and quantitative real-time PCR (qRT-PCR) analysis of *LrbHLH* genes

RNA-seq data for the *LrbHLH* genes were obtained from previous studies on gene expression in different flower developmental stages (Wang et al., 2021) and MeJA treatment (Wang et al., 2017). *LrbHLH* gene expression profiles were evaluated utilizing the values of FPKM. TBtools (Chen et al., 2020) software was utilized to generate *LrbHLH* expression heatmaps. Total RNA was extracted with RNAprep Pure Plant Kit (TIANGEN, Beijing, China) according to the manufacturer’s protocol. cDNA was synthesized by using PrimeScript™ II 1st Strand cDNA Synthesis Kit (TaKaRa Bio, Dalian, China) and utilized for qRT-PCR assays. Relative expression levels of genes were analyzed using qRT-PCR with SYBR^®^ Premix Ex Taq™ II (Takara Bio, Dalian, China) on a Bio-Rad iQ5 Real-Time PCR System (Bio-Rad, CA, USA) in 15 μL reactions. Each reaction contained 7.5 μL 2×TransStart^®^ Top Green qPCR SuperMix, 5.9 μL ddH_2_O, 1μL cDNA, and 0.6 μL of 10 μM forward and reverse primers. The RT-qPCR protocol included the following: the PCR reaction conditions were at 95°C for 5 min; denaturation 5 s at 95°C; 60°C for 30 s; 40 cycles. The *LrTIP41* gene was utilized as an endogenous control to normalize relative expression levels based on the 2^-ΔΔCt^ method (Schmittgen and Livak, 2008). The specific primer sequences used in this research are listed in **Supplementary Table 1**.

### Gene cloning and construction of expression vectors

Cloning of *LrbHLHs* was based on putative ORFs of unigenes from the RNA-seq database. Primers (**Supplementary Table 1**) were synthesized for ORF sequence amplification using Tks GflexTM DNA Polymerase (Takara Bio, Dalian, China) from *L. radiata* petal cDNA. Reaction conditions were: 5 min of 95°C, 35 cycles for 30 s at 94°C, 30 s at 60°C, 1 min at 72°C, with extension at 72°C for 10 min. PCR products were cloned into pMD19-T simple vectors (Takara Bio, Dalian, China). Afterward, those T-vectors were transferred into DH5α competent cells (Takara Bio, Dalian, China) for colony PCR amplification and then validated by sequencing.

### Subcellular localization analysis of LrbHLH proteins

Subcellular localization of LrbHLH proteins was predicted using WoLF PSORT (https://wolfpsort.hgc.jp; Horton et al., 2021), ProtComp 9.0 (http://linux1.softberry.com), and Plant-mPLoc (http://www.csbio.sjtu.edu.cn/bioinf/plant-multi/). The coding region of each *LrbHLH* gene was PCR-amplified with specific primers (**Supplementary Table 1**) and inserted into the plant expression vector pBinGFP4 (digested with KpnI/BamHI) with ClonExpress^®^ II One Step Cloning Kit (Vazyme, Nanjing, China). The recombinant vectors were then transformed into *Agrobacterium tumefaciens* EHA105 competent cells. The *A. tumefaciens* strains containing various constructs were cultivated, harvested, followed by resuspension in an invasive solution (10 mM MES, 0.2 mM Acetosyringone, and 10 mM MgCl_2_) with a final OD_600_ value of 0.6. Forty-day-old *Nicotiana benthamiana* plants were used for infiltration. After infiltration, plants were grown at 22°C in the dark and then transferred to normal growth conditions (25°C/16 h light and 22°C/8 h dark cycle) for three days. GFP fluorescent signals in the epidermal cells of *N. benthamiana* leaves were observed under a confocal microscope (Zeiss LSM900, Jena, Germany).

### PPI network prediction of LrbHLH proteins

Potential PPI network was predicted using STRING server online database based on *A. thaliana* homologous proteins (https://cn.string-db.org; Szklarczyk et al., 2021). The protein sequences of 50 LrbHLHs and 7 LrJAZs were uploaded into the server selecting *A. thaliana* as the comparative organism. The *LrbHLHs* genes interaction network was constructed after blasting with the highest bitscore.

### Analysis of LrbHLHs interactions utilizing yeast two-hybrid (Y2H) assay

The full-length coding regions (CDs) of *LrbHLH28, LrbHLH31*, *LrbHLH48* and *LrJAZs* (**Supplementary Table 4**) were cloned into pGADT7 (AD) or pGBKT7 (BD) vector, using the ClonExpress^®^ II One Step Cloning Kit (Vazyme, Nanjing, China) respectively. The auto-activation potentiality and interaction assay of LrbHLH and LrJAZ proteins were tested according to the Matchmaker Gold Y2H System (Takara Bio, Dalian, China). The positive clones were then sequentially selected on SD/-Ade/-His/-Leu/-Trp medium with 5-bromo-4-chloro-3-indoxyl α-D-galactoside (X-α-gal) and Aureobasidin A (AbA) to test the interactions between LrbHLHs and LrJAZs.

### Statistical analysis

All experiments were independently duplicated at least three times. The results were represented as mean ± SD of biological triplicates. The student’s t-test was utilized for data significance analysis at *p* < 0.05 or *p* < 0.01 level.

## Results

### Identification and characterization of LrbHLH proteins in *L. radiata*

We used HMMER 3.0 and BLASTP to predict putative LrbHLH protein sequences in the *L. radiata* transcriptome database with an E-value threshold of < 1 e^-5^ (Wang et al., 2021). All candidate sequences were confirmed in NCBI and with SMART to further identify conserved complete bHLH domains. In total, 50 LrbHLH proteins (namely LrbHLH1-LrbHLH50) were identified. The CDs of *LrbHLH* genes varied from 258 bp to 1,662 bp. LrbHLH proteins ranged from 86 to 554 amino acids in size with molecular weight of 9.75 kDa to 74.73 kDa and isoelectric point of 4.74 to 9.30. Subcellular localization prediction showed that most LrbHLH proteins was localized in the nucleus, and few LrbHLH proteins located in the cytoplasm, chloroplast, or mitochondria (**Supplementary Table 2a**).

### Phylogenetic analysis and classification of LrbHLH proteins

**Figure 1:**
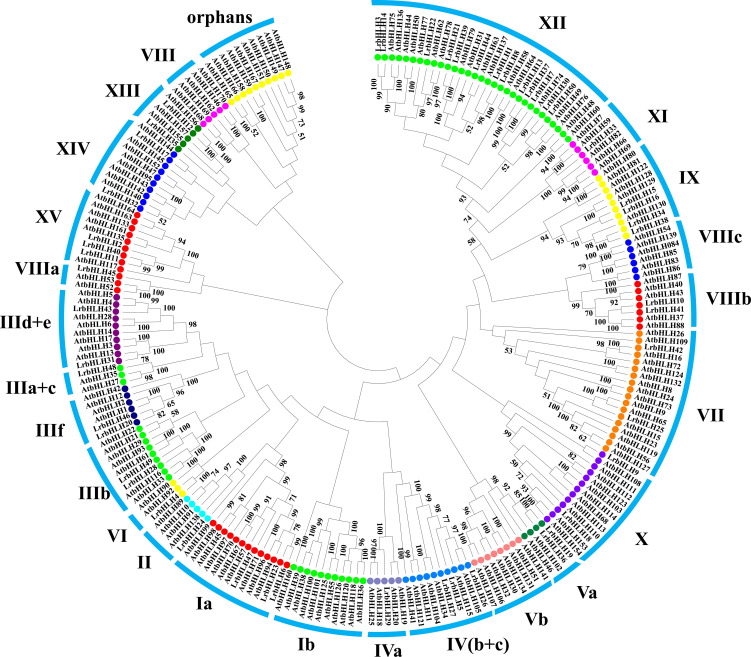
The phylogenetic analysis of *L. radiata* and *Arabidopsis* bHLH proteins. The neighbor-joining method was utilized to generate the phylogenetic tree relying on the bHLH domains alignment. These numbers are computed using 1000 bootstrap replicates to verify reliability. The replicate trees percentages over 50 are presented on branches. The tree shows the 25 subfamilies marked with blue font on a colored background.

Phylogenetic tree was constructed with LrbHLHs and *A. thaliana* bHLH (AtbHLH) protein sequences to investigate their evolutionary relationships. Fifty LrbHLH proteins were clustered into 20 groups, which were designated as group Ia, II, IIIa+c, IIIb, IIId+e, IIIf, IVa, IV(b+c), Va, VI, VII, VIIIa, VIIIb, IX, X, XI, XII, XIII, XIV, and XV (**Figure 1**). Among them, group XII was the largest group containing 13 LrbHLH proteins. In contrast, only one LrbHLH was found in group II, IIIa+c, IVa, VI, VIIIa, and XI. None of LrbHLH protein was found in group orphans, VIII, Ib, Vb, and VIIIc.

### Multiple sequence alignment, conserved motif, and bHLH domain analysis of LrbHLH proteins

**Figure 2:**
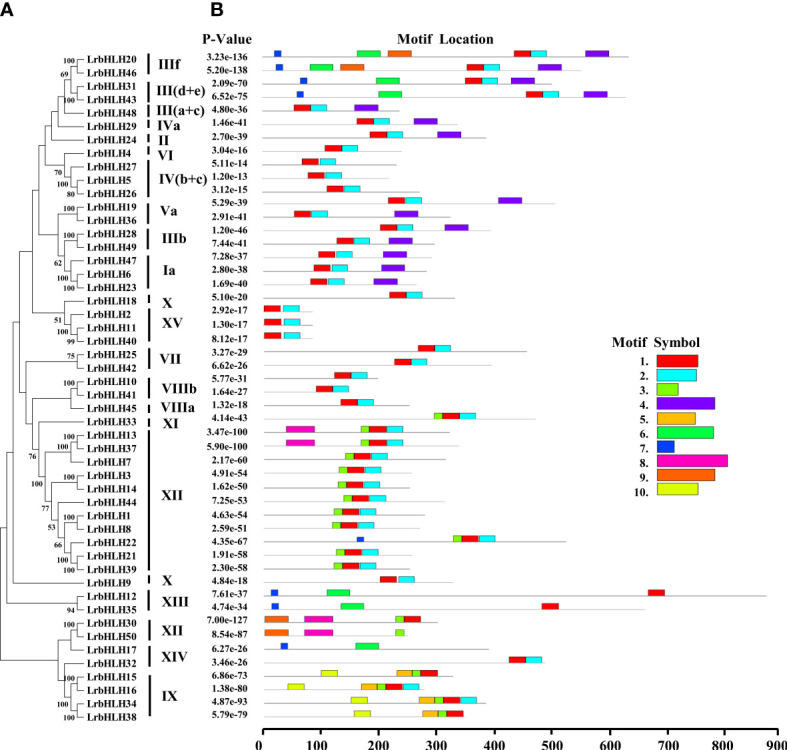
The phylogenetic relationships and conserved motifs analysis of LrbHLH proteins. **(A)** Neighbor-joining LrbHLHs phylogenetic tree (bootstrap values for 1000 replicates); **(B)** Conserved motifs distribution in LrbHLH proteins. Different motifs are represented with different colored boxes. The motif length is represented by the box length.

**Figure 3:**
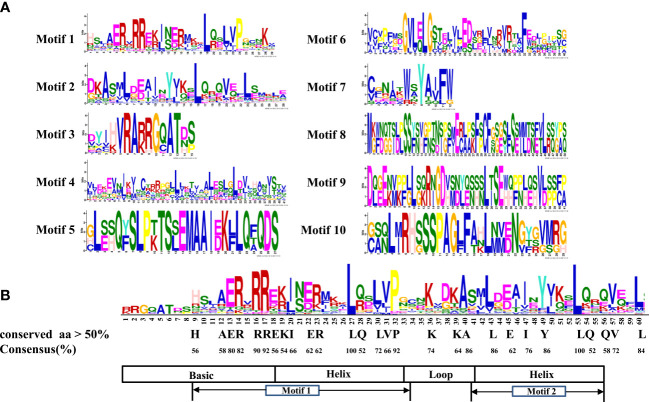
The conserved motifs analysis of LrbHLH proteins. **(A)** Conservation and diversity of the motifs in LrbHLH proteins. The schematic representation of ten motifs in LrbHLH proteins is elucidated by MEME; **(B)** LrbHLH proteins share a conserved bHLH domain. The amino acid height represents the sequence conservation. More than 50% amino acid conservation was presented with the capital letters, and the conserved amino acid percentages among bHLH domains are presented with numbers.

The conserved motifs of LrbHLH proteins were further predicted, and a total of 10 conserved motifs were found (**Figures 2**, **3A**). As shown in **Figure 2**, motif 1 was the most highly conserved motif present in almost all the LrbHLH proteins except LrbHLH17 and LrbHLH50. Motif 2 was the second most highly conserved motif. In addition, analysis of conserved amino acid residues in the bHLH domain showed that there are two helix regions, one basic region, and one loop region in LrbHLH proteins (**Figure 3B** and **Supplementary Figure 1**). Motif 1 was closer to the N-terminal and comprised with the basic region and the first helix region, whereas motif 2 mainly existed in the second helix region (**Figure 3B**). Moreover, motifs in LrbHLH proteins belonging to the same group were the same or structurally similar. For instance, motif 1, motif 2, and motif 3 were identified in all 11 LrbHLH proteins belonging to group XII. Motif 1, motif 2, motif 3, motif 5, and motif 10 were recognized in 4 LrbHLH proteins of group IX (**Figure 2**).

On the other hand, multiple sequence alignment of 50 LrbHLHs revealed that 28 amino acid residues were considerably conserved with a consensus ratio > 50%, and 11 of them were conserved with a consensus ratio > 75% (**Figure 3B** and **Supplementary Figure 1**). These 28 conserved residues were distributed among the basic regions (His-9, Ala-12, Glu-13, Arg-14, Arg-16, and Arg-17), the first helix region (Glu-18, Lys-19, Ile-20, Glu-22, Arg-23, Gln-28, Leu-27, Leu-30, Val-31, and Pro-32), the loop region (Lys-36 and Lys-39), and the second helix region (Ala-40, Leu-43, Glu-45, Ile-47, Tyr-49, Leu-53, Gln-54, and Gln-56). The DNA binding bHLH proteins were further categorized according to the residues Glu-13 and Arg-17 presence or absence in the basic regions with putative E-box and non-E-box binding proteins. Among LrbHLHs, 28 proteins containing His-9, Glu-13, Arg-14, Arg-16, and Arg-17 residues were confirmed to binding the G-box motif (CACGTG), whereas 12 LrbHLH proteins containing Glu-13 and Arg-17 residues were found to recognize the types of E-boxes (CANNTG) and were defined as non-G-box binders (**Figure 3B** and **Supplementary Figure 1**).

### The GO and KEGG enrichment between *LrbHLH* genes

**Figure 4:**
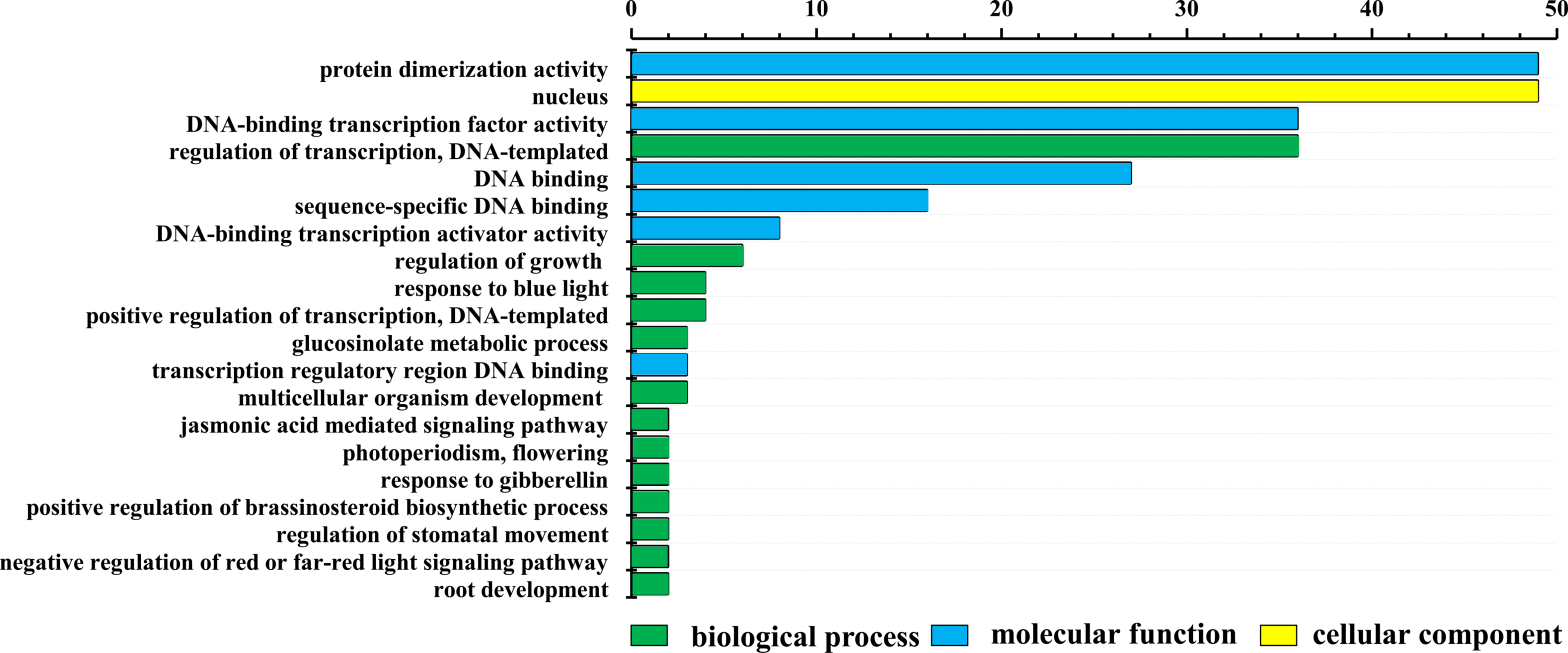
Gene ontology (GO) enrichment analysis of LrbHLH proteins. The top 20 GO terms of level 2 in biological process, cellular component, and molecular function were visualized. The X and Y axes represent the enriched protein numbers and the information on GO terms, respectively.

LrbHLH proteins were annotated to three main GO categories, including 36 biological process terms, 6 molecular function terms, and 4 cellular component terms. The top 20 GO terms of level two were visualized (**Figure 4**). In biological process terms, 36 LrbHLH proteins were implicated in the term ‘regulation of transcription, DNA-templated’. In cellular component terms, 49 LrbHLH proteins were components of the term ‘nucleus’, but few of them were components of the term ‘cytosol and cytoplasm’. In molecular function terms, 49 LrbHLH proteins and 36 LrbHLH proteins were categorized as exhibiting ‘protein dimerization activity’ and ‘DNA-binding transcription factor activity’, respectively (**Figure 4**).

The KEGG pathway analysis showed that LrbHLH25, LrbHLH32, and LrbHLH43 were annotated to three KEGG homologous proteins and participated in three pathways. LrbHLH25 was homologous to PIF4 (KO: K16189) and implicated in plant hormone signal transduction pathway (ko04075). The LrbHLH32 was a homolog of PIF3 (KO: K12126) and participated in circadian rhythm-plant pathway (ko04712) and plant hormone signal transduction pathway (ko04075). LrbHLH43 was annotated to MYC2 (KO: K13422) that participated in plant hormone signal transduction pathway (ko04075) and the MAPK signaling pathway (ko04016). Therefore, PIF3, PIF4 and MYC2 are all involved in plant hormone signal transduction pathway. PIF3 and MYC2 mainly transmit gibberellin (GA) signal pathway and JA signal pathway, respectively.

### Subcellular localization of LrbHLH proteins

**Figure 5:**
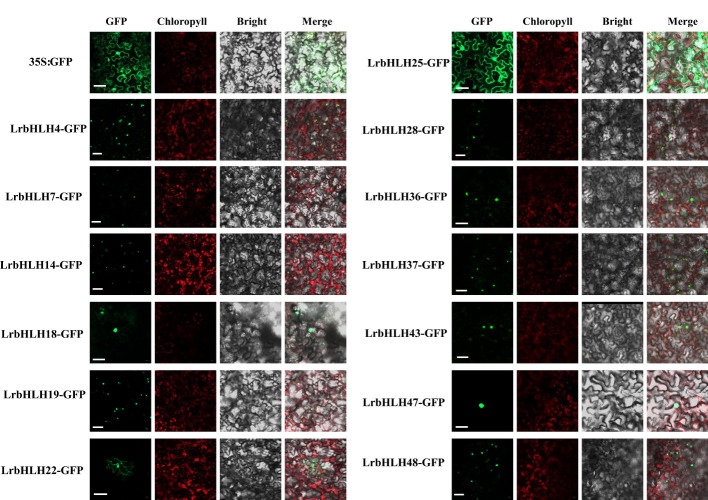
Subcellular localization of 15 LrbHLHs with GFP as a control, which was transiently expressed in *N. benthamiana* leaves. The photographs were taken using the green channel (GFP fluorescence), red channel (Chlorophyll represents chlorophyll auto fluorescence), bright channel, and their combination under a confocal microscope. Scale bar = 10 µm.

Most LrbHLH proteins were predicted to be localized in the nucleus, whereas few of LrbHLHs were located in the cytoplasm and/or other organelles (**Supplementary Table 2a**). We then examined the subcellular localization of some LrbHLH proteins in *N. benthamiana* epidermal cells. As expected, LrbHLH4, LrbHLH7, LrbHLH14, LrbHLH18, LrbHLH19, LrbHLH28, LrbHLH36, LrbHLH37, LrbHLH43, LrbHLH47, and LrbHLH48 were localized in the nucleus, whereas LrbHLH22 were located in both the nucleus and membrane, and LrbHLH25 was present in the nucleus and cytoplasm (**Figure 5**).

### Expression patterns analysis of *LrbHLH* genes in different tissues and at different flower developmental stages

**Figure 6:**
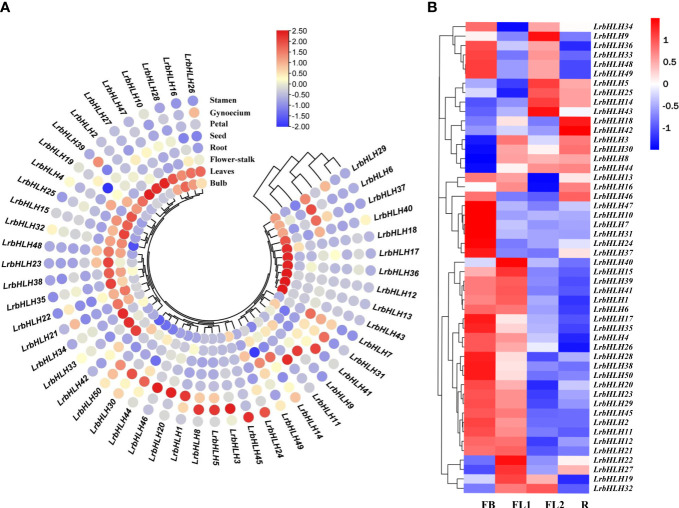
*LrbHLH* gene expression patterns in various *L. radiata* tissues. **(A)** Expression profile heatmap with hierarchal clustering of *LrbHLHs* in different tissues of *L. radiata*; **(B)** Expression profile heatmap with hierarchal clustering of *LrbHLHs* at different flower developmental stages of *L. radiata*. The relative expression for each genes is depicted by color intensity in each field. Higher values are represented by red whereas lower values are represented by blue. FB, flower-bud differentiation stage; FL1, partially opening flower stage; FL2, fully opened flower stage; R, senescent flower stage.

As the transcriptome analysis of different tissues (root, leaf, and bulb) has been revealed in *L. longituba* (Li et al., 2020a), we then searched the orthologous genes of *LrbHLH* by using local blast within *L. longituba* transcriptome data. 50 orthologous genes of *LrbHLH* were found in *L. longituba* (**Supplementary Table 2B**). As indicated in **Supplementary Figure 2**, some *LrbHLH* genes were exhibited differential expression in the three tissues, whereas other *LrbHLH* genes showed similar expression patterns in diverse tissues. For example, four *LrbHLHs* including *LrbHLH14*, *LrbHLH25*, *LrbHLH32*, and *LrbHLH44* were relatively highly expressed in leaves, whereas *LrbHLH5*, *LrbHLH12*, and *LrbHLH17* were preferentially expressed in roots. *LrbHLH6*, *LrbHLH39* and *LrbHLH49* had the highest relative expression levels in bulb. To further elucidate the biological function of LrbHLH proteins, qRT-PCR was utilized to determine the spatial specificity expression pattern of 50 *LrbHLH* genes in eight *L. radiata* organs. As indicated in **Figure 6A**, some *LrbHLH* genes were exhibited differential expression in the eight tissues, whereas other *LrbHLH* genes showed similar expression patterns in diverse tissues, which could be attributed to the functional differentiation of *LrbHLH* genes during plant development. For example, eight *LrbHLHs* (i.e., *LrbHLH2, LrbHLH4, LrbHLH15, LrbHLH16, LrbHLH25, LrbHLH26, LrbHLH27*, and *LrbHLH47*) were relatively highly expressed in leaves. *LrbHLH1*, *LrbHLH20*, *LrbHLH41* and *LrbHLH46* were preferentially expressed in petals. *LrbHLH3*, *LrbHLH5* and *LrbHLH8* showed highly expression levels in gynoecium, whereas *LrbHLH24* and *LrbHLH45* had relatively high expression levels in stamen. In addition, six *LrbHLHs* (i.e., *LrbHLH23*, *LrbHLH33*, *LrbHLH34*, *LrbHLH35*, *LrbHLH38* and *LrbHLH42*) were highly expressed in flower-stalk tissue. In particular, *LrbHLH6*, *LrbHLH7*, *LrbHLH14* and *LrbHLH37* were predominantly expressed in root. *LrbHLH9* and *LrbHLH11* were relatively highly abundant in seed. *LrbHLH12, LrbHLH13, LrbHLH17, LrbHLH18, LrbHLH36, LrbHLH40 and LrbHLH43* had the highest relative expression levels in bulb. Conversely, some genes were not expressed specifically. Furthermore, *LrbHLH29*, *LrbHLH49* and *LrbHLH50* showed low expression in all tissues. These results suggested that *LrbHLHs* might play the same role in the growth and development of *L. radiata*. Additionally, most *LrbHLHs* exhibited diverse tissue-specific expression patterns, implying their numerous functions in diverse organs.

The expression of *LrbHLHs* was further observed at four flower development stages including flower-bud differentiation (FB) stage, partially opening flower (FL1) stage, fully opened flower (FL2) stage, and senescent flower (R) stage, based on our previously published RNA-seq data (**Figure 6B**, Wang et al., 2021). Over half of *LrbHLH* genes was highly expressed at FB stage and then markedly decreased at R stage. In addition, the expression of *LrbHLH8* and *LrbHLH44* exhibited a gradual increase during flower development. Remarkably, the expression of *LrbHLH5*, *LrbHLH14*, *LrbHLH25* and *LrbHLH43* genes exhibited opposite trends in the flowering developmental stages, indicating they may perform diverse functions. However, the expression of *LrbHLH18* and *LrbHLH42* were relatively higher at the late stages of flower development.

### Expression patterns of *LrbHLHs* in response to MeJA treatment

**Figure 7:**
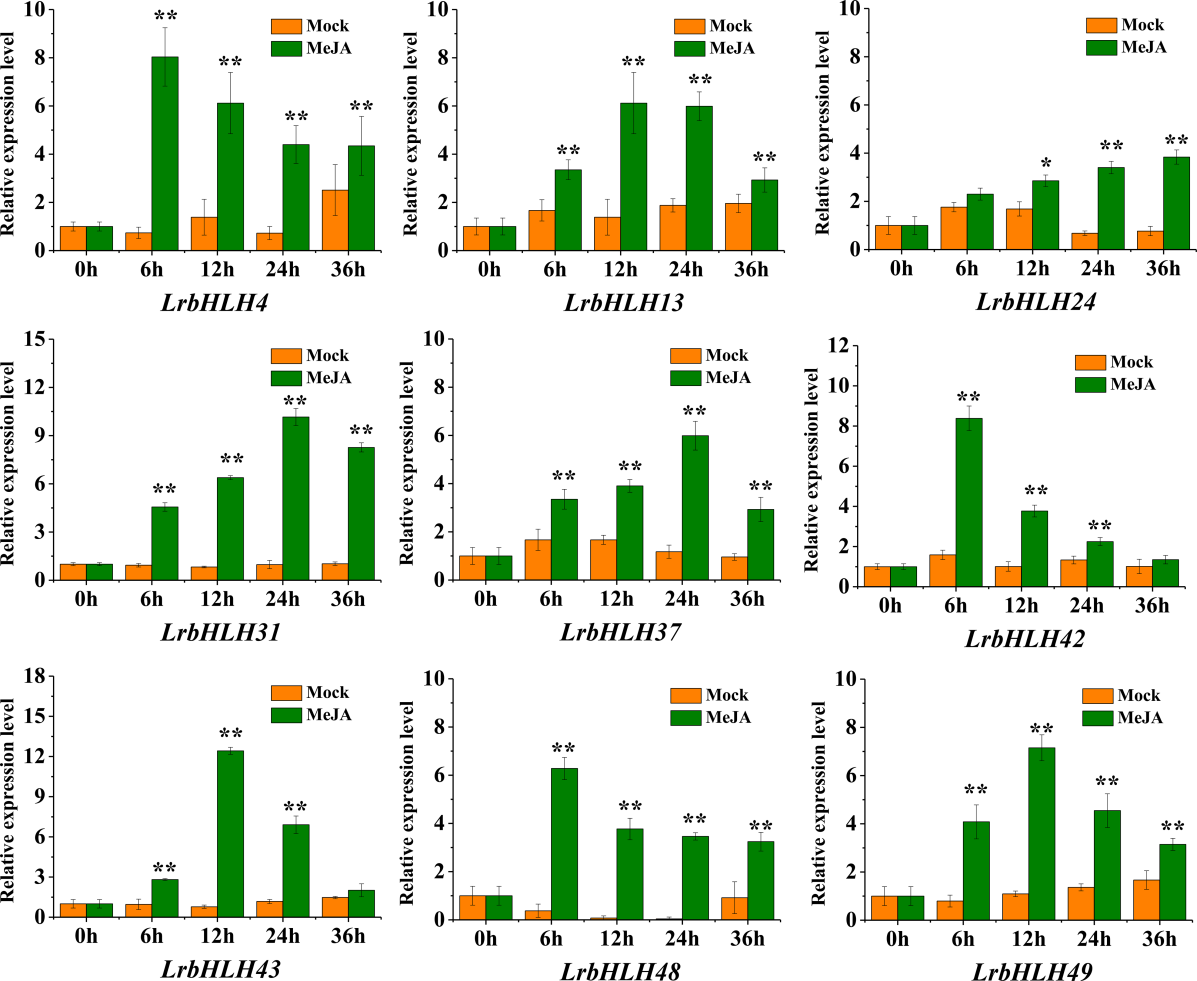
qRT-PCR analysis of *LrbHLHs* under MeJA treatment. The values represent means ± SD (n = 3). * and ** indicates significant difference between control and treatment according to student’s t-test at *p* < 0.05 and *p* < 0.01 level, respectively.

Previous study of *L. aurea* transcriptome sequencing has revealed that MeJA treatment could induce the expression of *LabHLH* genes (Wang et al., 2017). Thus, the orthologous genes of *LrbHLH* in *L. aurea* transcriptome data were also searched. All the 50 *LrbHLH* genes were found to have orthologous transcripts in *L. aurea* transcriptome (**Supplementary Table 2C**). Among them, 22 homologous *LrbHLHs* were up-regulated, while 10 homologous *LrbHLH* genes were suppressed with MeJA treatment for 6 h (**Supplementary Figure 3**). In addition, homologous genes of *LrbHLH6*, *LrbHLH23*, *LrbHLH34*, *LrbHLH35*, and *LrbHLH38* exhibited no expression changes after MeJA treatment. Furthermore, 9 *LrbHLH* genes including *LrbHLH4*, *LrbHLH13*, *LrbHLH24*, *LrbHLH31*, *LrbHLH37*, *LrbHLH42*, *LrbHLH43*, *LrbHLH48* and *LrbHLH49* were subjected to qRT-PCR for confirming their expression profiles under MeJA with different treatment time in *L. radiata* roots. Consequently, these *LrbHLHs* were significantly up-regulated, which were similar to their expression trends in RNA-Seq data (**Figure 7**). These findings revealed that *LrbHLHs* displayed different expression patterns in response to MeJA treatment and potentially participate in biological processes *via* JA signaling pathways.

### PPI networks of LrbHLH proteins

**Figure 8:**
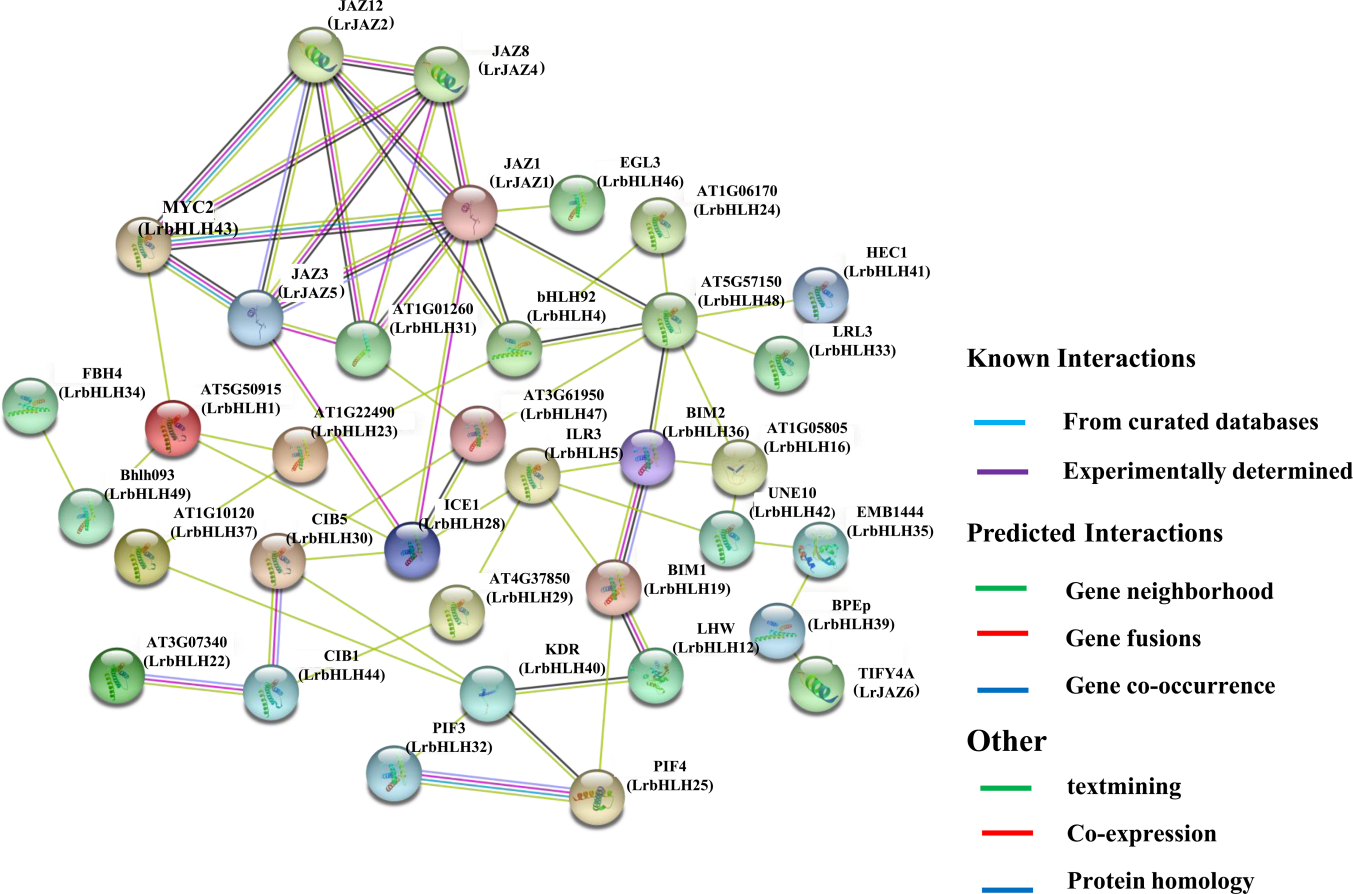
The predicted network of protein-protein interactions between LrbHLHs by STRING database. Different colors represent different interaction types. *Arabidopsis* bHLH names are marked, whereas their homologs in *L. radiata* are in parentheses.

Based on the protein orthologs of *Arabidopsis* (**Supplementary Table 3** and **Table 4**), LrbHLH proteins and LrJAZ proteins homologous to LaJAZs (Wang et al., 2020) were analyzed to determine functional and physical interactions using the STRING database (**Supplementary Table 5**). Generally, several key interactions were predicted, and some LrbHLH and LrJAZ proteins might interact with at least four proteins (**Figure 8**). For instance, LrbHLH48 (homologous to At5g57150) can interact with many LrbHLH proteins including LrbHLH4 (homologous to AtbHLH92), LrbHLH16 (homologous to At1g05805), LrbHLH24 (homologous to At1g06170), LrbHLH33 (homologous to AtLRL3), LrbHLH36 (homologous to AtBIM2), LrbHLH41 (homologous to AtHEC1), LrbHLH47 (homologous to At3g61950) and LrJAZ1 (homologous to AtJAZ1), acting as a center of the network node. Meanwhile, LrJAZ1 could also function as another center of the network node, showing the multiple interactions with LrbHLH and LrJAZ proteins. These findings confirm the functional diversity of *LrbHLH* genes.

**Figure 9:**
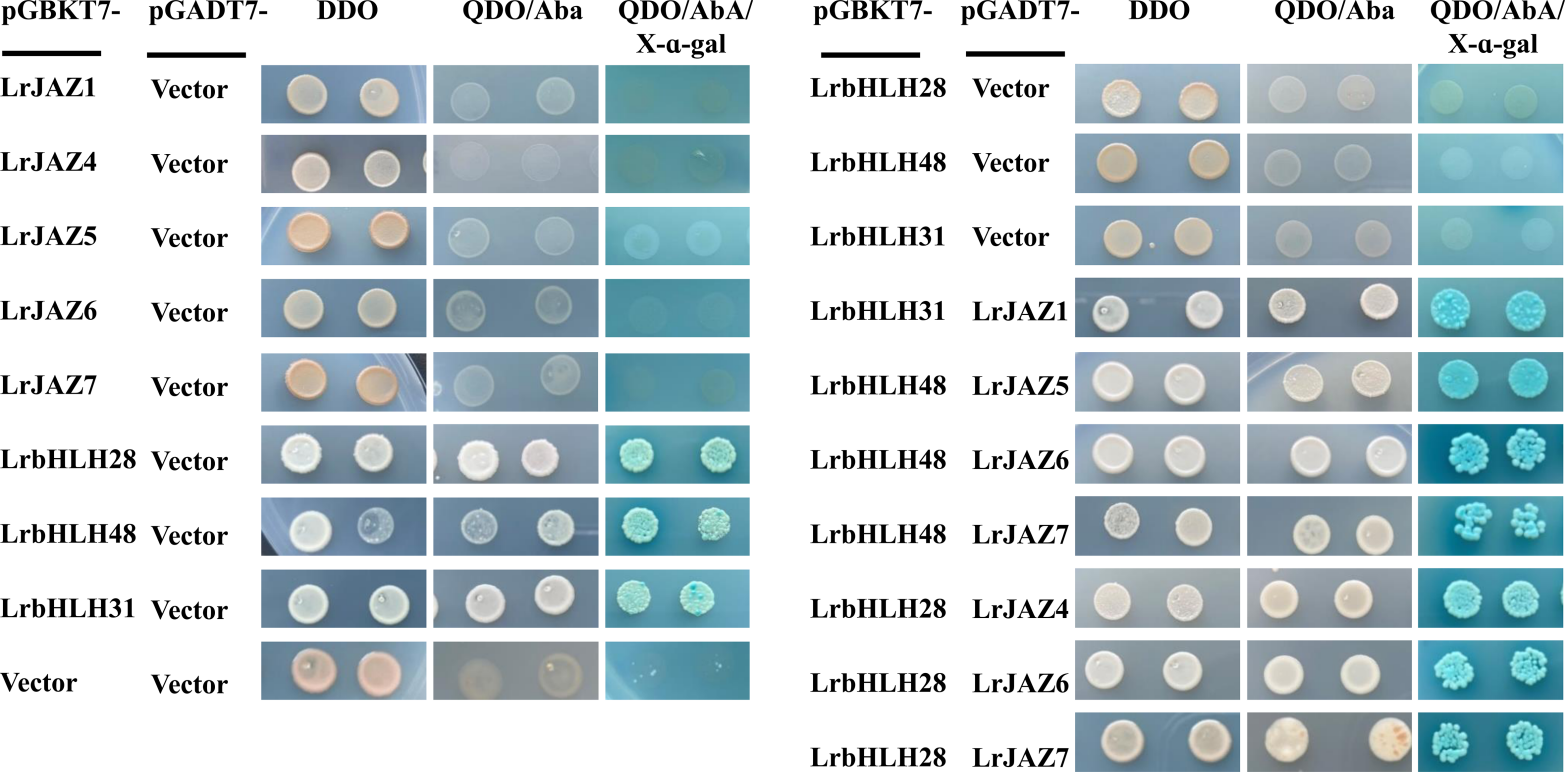
The protein-protein interactions between LrbHLHs and LrJAZs detected by yeast two-hybrid (Y2H) system. The coding sequences of *LrbHLH28*, *LrbHLH31* and *LrbHLH48* were ligated to the GAL4 activation domain (AD) and DNA-binding domain (BD), whereas the sequences of *JAZ* genes were fused to the GAL4 activation domain (AD). Positive control, negative control, and the fusion constructs were transformed into the Y2H strain and successively incubated in DDO (SD/-Leu/-Trp) medium, QDO (SD/-Ade/-Leu/-Trp/-His) medium supplemented with Aureobasidin A (AbA) and QDO medium supplemented with 5-bromo-4-chloro-3-indoxyl α-D-galactoside (X-α-Gal) and AbA.

To further verify protein-protein interactions in the predicted network, we randomly selected three *LrbHLH* (*LrbHLH28*, *LrbHLH31* and *LrbHLH48*) genes and five *LrJAZ* (*LrJAZ1*, *LrJAZ4*, *LrJAZ5*, *LrJAZ6* and *LrJAZ7*) genes to investigate their interactions using the Y2H technique in yeast (**Figure 9**). None of the three LrbHLH proteins could interact with the empty BD vector, while these LrbHLH proteins could interact with the empty AD, suggesting their self-activation. In addition, none of LrJAZ1, LrJAZ4, LrJAZ5, LrJAZ6, and LrJAZ7 proteins could interact with AD empty vector, indicating they have no self-activation (**Figure 9**). Among the combination of LrbHLH proteins and LrJAZ proteins, LrbHLH31 and JAZ1 showed a strong interaction. In addition, LrbHLH28 could interact with JAZ4, JAZ6, and JAZ7; whereas LrbHLH48 could interact with JAZ5, JAZ6, and JAZ7 respectively.

## Discussion

### Characterization of the *bHLH* gene family in *L. radiata*

In plants, bHLH TF family is the second largest TFs family. For example, there are 162 bHLHs identified in *A. thaliana* (Carretero-Paulet et al., 2010), 167 bHLHs in rice (Carretero-Paulet et al., 2010), 230 bHLHs in *Brassica rapa* (Song et al., 2014), and 319 bHLHs in *Glycine max* (Hudson and Hudson, 2015). In this study, we identified 50 *LrbHLH* candidate genes through homolog search and domain analysis utilizing previously published transcriptome data (**Supplementary Table 2**; Wang et al., 2021). The *LrbHLHs* had similar properties in terms of the encoding amino acids number, isoelectric point, and motifs to bHLHs in *A. thaliana* and other plants (Carretero-Paulet et al., 2010). Plant bHLH members were grouped into several clades (or subfamilies), with the numbers of clades varying from 15 to 25 (Heim et al., 2003; Carretero-Paulet et al., 2010). We also found that 50 LrbHLH proteins in *L. radiata* could be classified into 20 clades (**Figure 1**). Interestingly, no LrbHLH proteins were in group orphans, VIII, Ib, Vb, VIIIc based on the *Arabidopsis* classification, showing *L. radiata* may lose homologs in these group throughout evolution. In addition, several LrbHLH proteins were grouped into functional clades of *Arabidopsis*, providing valuable insights for studying their biological roles. For example, LrbHLH20 and LrbHLH46 were grouped into subfamily IIIf and showed highly homologous to *Arabidopsis* MYC1, TT8, GLABROUS3 (GL3), and ENHANCER OF GLABRA3 (EGL3) proteins. It has been proved that AtTT8, AtGL3, and AtEGL3 could regulate anthocyanin and proanthocyanidins (PA) biosynthesis in *Arabidopsis* (Li, 2014). Besides, AtGL3 and AtEGL3 could modulate root hair patterning and trichome formation (Ramsay and Glover, 2005; Zhao et al., 2012). Thus, LrbHLH20 and LrbHLH46 might potentially modulate PA biosynthesis and trichome formation in *L. radiata*.

The conserved bHLH domain with estimated 60 amino acids comprised one basic, two helixs, and one loop regions (Li et al., 2006). This structure was also observed in *L. radiata* (**Figure 3**). Nineteen amino acid residues in the LrbHLH proteins are highly conserved, with a consensus ratio over 50%. These residues are highly conserved in bHLH TFs of *Arabidopsis*, *Zea mays*, and grape, suggesting the conservation of bHLH TFs families among different plants (Toledo-Ortiz et al., 2003; Zhang et al., 2018a; Wang et al., 2018). In addition, at least five basic residues in the basic region of the bHLH domain determine their DNA binding activity of bHLH TFs, and Glu-13 is important in specific recognition of the E-box DNA binding motif, whereas Arg-16 fixes and stabilizes the Glu-13 position. Moreover, His/Lys-9 and Arg-17 confer peculiarity to G-box discriminates, while Leu-27 is critical for dimer formation (Toledo-Ortiz et al., 2003). We also observed that Leu-27 was conserved in all LrbHLHs and homologous with AtbHLH proteins. Those results indicated that each LrbHLH protein have the basic functional property.

### The GO annotation of *LrbHLH* genes indicating their potential functions

These enriched GO annotations of *LrbHLH* genes match to the known functional bHLH TFs. For instance, *LrbHLH* genes were predominantly involved in the ‘DNA binding transcription factor’, ‘transcription regulator’, ‘DNA binding’, and ‘protein dimerization activities’ in the molecular function section (**Figure 4**). This conformed that bHLH TFs regulate numerous genes implicated in different regulatory pathways (Castelain et al., 2012). For example, bHLH TFs NtMYC2a and NtMYC2b could form nuclear complexes with the NtJAZ1 repressor and modulate multiple jasmonate-inducible steps in nicotine biosynthesis (Zhang et al., 2012). bHLH TFs could also respond to Fe-deficient and salinity stresses (Huang and Dai, 2015; Babitha et al., 2015). *TcMYC* is highly expressed in leaves and xylem, unregulated by high-salinity and drought stresses in yew trees (Yang et al., 2018). The *CsbHLH18* of sweet orange regulates the antioxidant gene, which helps in the modulation of cold tolerance (Geng and Liu, 2018).

### The *bHLH* gene expression patterns facilitate their functional analysis

We systematically analyzed the expression patterns of *LrbHLH* genes in various tissues to investigate their physiological role in *L. radiata* biological processes. The homologous genes with similar expression profiles are conserved due to the dosage effect, whereas homologous genes with different expression profiles are retained by functionalization and subfunctionalization (Kim et al., 2014). We also observed that *LrbHLH3*/*LrbHLH14*, *LrbHLH21*/*LrbHLH39*, *LrbHLH1*/*LrbHLH8*, *LrbHLH13*/*LrbHLH37*, *LrbHLH30*/*LrbHLH50*, *LrbHLH15*/*LrbHLH16*, *LrbHLH34*/*LrbHLH38*, *LrbHLH10*/*LrbHLH41*, *LrbHLH19*/*LrbHLH36*, *LrbHLH6*/*LrbHLH23*, *LrbHLH20*/*LrbHLH46* and *LrbHLH11*/*LrbHLH40* were homologous genes (**Figure 1**). Among them, two gene pairs including *LrbHLH20*/*LrbHLH46* and *LrbHLH30*/*LrbHLH50* showed similar expression pattern, whereas other ten duplicated pairs had significantly different expression profiles in different tissues (**Supplementary Figure 2**; **Figure 6**). This suggests that there was a functional divergence among duplicated LrbHLH pairs at some point during evolution process. In addition, several *LrbHLH* genes highly expressed in specific tissues suggested their potential roles in organ development. For instance, *LrbHLH1*/*LrbHLH8* were homolog to *AtbHLH31*, which participates in targeting cell expansion during petal growth (Varaud et al., 2011) exhibited higher expression level during flower development, suggesting *LrbHLH1*/*LrbHLH8* may have the similar function in *L. radiata*. Meanwhile, we identified one putative *MYC2* homolog gene (Dombrecht et al., 2007) *LrbHLH43*, which only exhibited higher expression level in bulb, suggesting its specific function in bulb. Otherwise, *bHLH* genes showed extensive expression levels in roots, including 78 *bHLH* genes in maize (Zhang et al., 2018a), five *bHLH* genes in wheat (Wang et al., 2019a), and two *bHLH* genes in *Dendrobium officinale* (Wang and Liu, 2020). We also observed that *LrbHLH6*, *LrbHLH7*, *LrbHLH14*, and *LrbHLH37* genes were highly expressed in roots (**Figure 6A**), implying they could play important roles in *L. radiata* roots.

JA is a critical defense signaling molecule regulating different biological processes. Previous study revealed that MeJA could regulate the expression of *bHLH* family genes (Zander et al., 2020; Guo et al., 2021; Li et al., 2022). Recently, tomato bHLH gene *SlJIG* has been demonstrated to be induced by JA, and function in terpene biosynthesis and resistance to insects and fungus (Cao et al., 2022). We also analyzed the expression levels of *bHLH* genes in *Lycoris* under MeJA treatment. Most of the *LrbHLH* genes and orthologous genes of *LrbHLH (LabHLH*) were simultaneously affected by MeJA treatments (**Supplementary Figure 3** and **Figure 7**). Those results indicate that the important biological functions of *LrbHLHs* may correlated with JA signaling pathway.

### PPI network prediction and validation of LrbHLHs

The PPI networks were utilized for identifying the interactions between *LrbHLHs*. bHLH TF MYC2 and its homologs MYC3 and MYC4 acts as the master regulator of diverse JA responses (Breeze, 2019). Meanwhile, the plant-specific group of JAZ proteins plays a critical part in repressing the activity of MYC TFs (Howe et al., 2018). Subsequently, we analyzed the potential interactions among LrbHLH proteins and JAZ proteins in the anticipated network. Consequently, LrbHLHs and JAZ proteins were found to form a signal transmission network (**Figure 8** and **Supplementary Figure 4**). This is consistent with former researches that the bHLH proteins binding activity is based on the homodimers or heterodimers formation among bHLH proteins (Pires and Dolan, 2010; Feller et al., 2011). FLOWERING BHLH 4 (FBH4, homolog of LrbHLH34 and LrbHLH38) and CIB1 (homolog of LrbHLH44) are implicated in the regulation of flowering time (Ito et al., 2012; Liu et al., 2013); HECATE (HEC, homolog of LrbHLH10 and LrbHLH41) interact with SPATULA (SPT) for the pistil development regulation through targeting cytokinins among other hormones (Schuster et al., 2015). SCREAM (SCRM, also known as ICE1; homolog of LrbHLH28) can interact with FAMA, SPEECHLESS (SPCH), and MUTE and regulate stomatal differentiation (Qi and Torii, 2018). Moreover, INDUCER OF CBP EXPRESSION 1 (ICE1) regulates lateral bud growth and plant stress response (Zhang et al., 2018b), whereas *Lotus japonicas* ROOTHAIRLESS LIKE (LRL3, homolog of LrbHLH33) regulates root hair development (Breuninger et al., 2016). MYC2 (homolog of LrbHLH43) is vital in the optical, GA, and JA signals as well as modulates many signal transmissions (Lorenzo et al., 2004; Dombrecht et al., 2007). PIF3 (homolog of LrbHLH32) interacts with phyB1 in light signal transduction (Kumar et al., 2016b). Additionally, LrbHLH25 may have the similar functions with PIF4 and PIF5 and play conserved roles in the response to phytochrome signaling in plants (Shi et al., 2018). Furthermore, we confirmed protein interactions between LrbHLH28, LrbHLH31, LrbHLH48, and LrJAZ proteins (**Figure 9**). *LrbHLH28* has a highly expression level at flower developmental stage and belong to ICE1 branch, which could regulate the growth of lateral bud (Zhang et al., 2018b). Meanwhile, ICE1 could interact with the HOS1 protein, a crucial flowering time regulator (Liu et al., 2016). Flower bHLH transcriptional activator FBH4 may control expression of the photoperiodic flowering regulator *CONSTANS* to regulate flowering (Ito et al., 2012). LrbHLH34 and LrbHLH38 were homologs of FBH4, suggesting they might cause early flowering regardless of the photoperiod. Additionally, CRY2-interacting bHLH (CIB) proteins such as CIB1, CIB2, CIB3, CIB4, and CIB5 could activate *Flowering Locus T* (*FT*) transcription through the interaction with cryptochrome 2 (CRY2) protein for mediating flowering time regulation in *Arabidopsis* (Liu et al., 2013). Since *CIB* genes are conserved in plants (Liu et al., 2013), LrbHLH44 (CIB1 homologs) and LrbHLH30 (CIB5 homologs) might have the similar function in flower development. Additional research is necessary to demonstrate the comprehensive interaction network of the LrbHLH TFs during *L. radiata* growth and development.

## Conclusion

In this study, 50 *LrbHLH* genes were identified from the transcriptome of *L. radiata*, and enriched by systematically analyzing basic biochemical information, phylogenetic relationships, conserved motifs, and gene expression profiling. Most *LrbHLH* genes were revealed to likely play various important roles in *Lycoris* growth and development, especially in flower development stages. The analysis of the expression pattern of *LrbHLH* genes in response to MeJA treatment suggested that the *LrbHLH* genes could be importantly involved in JA signaling. Meanwhile, by comprehensive analysis of the comparison of homologous functional proteins, the PPI prediction networks and the yeast two-hybrid system, LrbHLH28, LrbHLH31 and LrbHLH48 was demonstrated to interact with LrJAZ proteins and might functionally regulate biological processes in JA signaling pathway. These findings provide a serviceable opportunity to understand the biological characteristics and functions of *LrbHLH* genes in *Lycoris*.

## Data availability statement

The datasets presented in this study can be found in online repositories. The names of the repository/repositories and accession number(s) can be found in the article/**Supplementary Material**.

## Author contributions

NW and ZW designed and conceived experiments, and wrote the manuscript. NW performed most of the experiments and data analysis. XS collected the experimental materials. FZ provided helpful comments on the manuscript. ZW provided guidance on the whole study and contributed with valuable discussions. All authors read and approved the final manuscript.

## Supplementary material

Supplementary Figure 1 Multiple sequence alignment of the bHLH conserved domain in *L.radiata* bHLH proteins. The yellow boxes indicate 30% identity of amino acids, the blue boxes indicate 50% identity of amino acids, and the red boxes indicate 75% identity of amino acids.

Supplementary Figure 2 Expression profile heatmap with hierarchal clustering of LrbHLHs in different tissues of *L. longituba.* Red and blue represent high and low relative transcript abundance, respectively.

Supplementary Figure 3 Heatmap of *LrbHLH* gene expression profiles with MeJA treatment. Red and blue represent high and low relative transcript abundance, respectively.

Supplementary Figure 4 The predicted network of protein-protein interactions between LrbHLHs by STRING database.

## References

[B1] Arnaud N. Girin T. Sorefan K. Fuentes S. Wood T. A. Lawrenson T.. (2010). Gibberellins control fruit patterning in *Arabidopsis thaliana* . Genes Dev. 24, 2127–2132. doi: 10.1101/gad.593410 20889713 PMC2947765

[B2] Artimo P. Jonnalagedda M. Arnold K. Baratin D. Csardi G. De Castro E.. (2012). ExPASy: SIB bioinformatics resource portal. Nucleic Acids Res. 40, W597–W603. doi: 10.1093/nar/gks400 22661580 PMC3394269

[B3] Babitha K. C. Vemanna R. S. Nataraja K. N. Udayakumar M. (2015). Overexpression of *EcbHLH57* transcription factor from *Eleusine coracana* l. @ in tobacco confers tolerance to salt, oxidative and drought stress. PLoS One 10, e0137098. doi: 10.1371/journal.pone.0137098 26366726 PMC4569372

[B4] Bailey T. L. Johnson J. Grant C. E. Noble W. S. (2015). The MEME suite. Nucleic Acids Res. 43, W39–W49. doi: 10.1093/nar/gkv416 25953851 PMC4489269

[B5] Baudry A. Heim M. A. Dubreucq B. Caboche M. Weisshaar B. Lepiniec L. (2004). TT2, TT8, and TTG1 synergistically specify the expression of BANYULS and proanthocyanidin biosynthesis in *Arabidopsis thaliana* . Plant J. 39, 366–380. doi: 10.1111/j.1365-313X.2004.02138.x 15255866

[B6] Breeze E. (2019). Master MYCs: MYC2, the jasmonate signaling “master switch”. Plant Cell 31, 9–10. doi: 10.1105/tpc.19.00004 30626620 PMC6391689

[B7] Breuninger H. Thamm A. Streubel S. Sakayama H. Nishiyama T. Dolan L. (2016). Diversification of a transcription factor family led to the evolution of antagonistically acting genetic regulators of root hair growth. Curr. Biol. 26, 1622–1628. doi: 10.1016/j.cub.2016.04.060 27265398 PMC4920954

[B8] Bu D. Luo H. Huo P. Wang Z. Zhang S. He Z.. (2021). KOBAS-i: intelligent prioritization and exploratory visualization of biological functions for gene enrichment analysis. Nucleic Acids Res. 49, W317–W325. doi: 10.1093/nar/gkab447 34086934 PMC8265193

[B9] Cahlíková L. Breiterová K. Opletal L. (2020). Chemistry and biological activity of alkaloids from the genus *Lycoris* (Amaryllidaceae). Molecules 25, 4797. doi: 10.3390/molecules25204797 33086636 PMC7587589

[B10] Cao Y. Liu L. Ma K. Wang W. Lv H. Gao M.. (2022). The jasmonate-induced bHLH gene *SlJIG* functions in terpene biosynthesis and resistance to insects and fungus. J. Integr. Plant Biol. 64, 1102–1115. doi: 10.1111/jipb.13248 35293128

[B11] Carretero-Paulet L. Galstyan A. Roig-Villanova I. Martínez-García J. F. Bilbao-Castro J. R. Robertson D. L. (2010). Genome-wide classification and evolutionary analysis of the bHLH family of transcription factors in *Arabidopsis*, poplar, rice, moss, and algae. Plant Physiol. 153, 1398–1412. doi: 10.1104/pp.110.153593 20472752 PMC2899937

[B12] Castelain M. Hir. R. Bellini C. (2012). The non-DNA-binding bHLH transcription factor PRE3/bHLH135/ATBS1/TMO7 is involved in the regulation of light signaling pathway in *Arabidopsis* . Physiol. Plant 145, 450–460. doi: 10.1111/j.1399-3054.2012.01600.x 22339648

[B13] Castillon A. Shen H. Huq E. (2007). Phytochrome interacting factors: central players in phytochrome-mediated light signaling networks. Trends Plant Sci. 12, 514–521. doi: 10.1016/j.tplants.2007.10.001 17933576

[B14] Chen C. J. Chen H. Zhang Y. Thomas H. R. Frank M. H. He Y. H.. (2020). TBtools: an integrative toolkit developed for interactive analyses of big biological data. Mol. Plant 13, 1194–1202. doi: 10.1016/j.molp.2020.06.009 32585190

[B15] Chen G. L. Tian Y. Q. Wu J. L. Li N. Guo M. Q. (2016). Antiproliferative activities of amaryllidaceae alkaloids from *Lycoris radiata* targeting DNA topoisomerase I. Sci. Rep. 6, 38284. doi: 10.1038/srep38284 27922057 PMC5138836

[B16] Dombrecht B. Xue G. P. Sprague S. J. Kirkegaard J. A. Ross J. J. Reid J. B.. (2007). MYC2 differentially modulates diversejasmonate-dependent functions in *Arabidopsis* . Plant Cell 19, 2225–2245. doi: 10.1105/tpc.106.048017 17616737 PMC1955694

[B17] Feller A. Machemer K. Braun E. L. Grotewold E. (2011). Evolutionary and comparative analysis of MYB and bHLH plant transcription factors. Plant J. 66, 94–116. doi: 10.1111/j.1365-313X.2010.04459.x 21443626

[B18] Feng T. Wang Y. Y. Su J. Li Y. Cai X. H. Luo X. D. (2011). Amaryllidaceae alkaloids from *Lycoris radiata* . Helv. Chim. Acta 94, 178–183. doi: 10.1002/hlca.201000176

[B19] Feng X. M. Zhao Q. Zhao L. L. Qiao Y. Xie X. B. Li H. F.. (2012). The cold-induced basic helix-loop-helix transcription factor gene *MdCIbHLH1* encodes an ICE-like protein in apple. BMC Plant Biol. 12, 22. doi: 10.1186/1471-2229-12-22 22336381 PMC3352023

[B20] Gao C. Sun J. Wang C. Dong Y. Xiao S. Wang X.. (2017). Genome-wide analysis of basic/helix-loop-helix gene family in peanut and assessment of its roles in pod development. PloS One 12, e0181843. doi: 10.1371/journal.pone.0181843 28750081 PMC5531549

[B21] Geng J. J. Liu J. H. (2018). The transcription factor CsbHLH18 of sweet orange functions in modulation of cold tolerance and homeostasis of reactive oxygen species by regulating the antioxidant gene. J. Exp. Bot. 69, 2677–2692. doi: 10.1093/jxb/ery065 29474667 PMC5920331

[B22] Gremski K. Ditta G. Yanofsky M. F. (2007). The HECATE genes regulate female reproductive tract development in *Arabidopsis thaliana* . Development 134, 3593–3601. doi: 10.1242/dev.011510 17855426

[B23] Guo J. Sun B. He H. Zhang Y. Tian H. Wang B. (2021). Current understanding of bHLH transcription factors in plant abiotic stress tolerance. Int. J. Mol. Sci. 22, 4921. doi: 10.3390/ijms22094921 34066424 PMC8125693

[B24] Heim M. A. Jakoby M. Werber M. Martin C. Weisshaar B. Bailey P. C. (2003). The basic helix-loop-helix transcription factor family in plants: a genome-wide study of protein structure and functional diversity. Mol. Biol. Evol. 20, 735–747. doi: 10.1093/molbev/msg088 12679534

[B25] Horton P. Park K. J. Obayashi T. Fujita N. Harada H. Adams-Collier C. J.. (2021). WoLF PSORT: protein localization predictor. Int. J. Mol. Sci. 22, 11458. doi: 10.3390/ijms222111458 17517783 PMC1933216

[B26] Howe G. A. Major I. T. Koo A. J. (2018). Modularity in jasmonate signaling for multistress resilience. Annu. Rev. Plant Biol. 69, 20.1–20.29. doi: 10.1146/annurev-arplant-042817-040047 29539269

[B27] Huang D. Q. Dai W. H. (2015). Molecular characterization of the basic helix-loop-helix (bHLH) genes that are differentially expressed and induced by iron deficiency in *Populus* . Plant Cell Rep. 34, 1211–1124. doi: 10.1007/s00299-015-1779-8 25721202

[B28] Hudson K. A. Hudson M. E. (2015). A classification of basic helix-loop-helix transcription factors of soybean. Int. J. Genomics 2015, 603182. doi: 10.1155/2015/603182 25763382 PMC4339708

[B29] Ito S. Song Y. Josephson-Day A. R. Miller R. J. Breton G. Olmstead R. G.. (2012). FLOWERING BHLH transcriptional activators control expression of the photoperiodic flowering regulator *CONSTANS* in *Arabidopsis* . Proc. Natl. Acad. Sci. U.S.A. 109, 3582–3587. doi: 10.1073/pnas.1118876109 22334645 PMC3295255

[B30] Jiang Y. Yang B. Deyholos M. K. (2009). Functional characterization of the arabidopsis bHLH92 transcription factor in abiotic stress. Mol. Genet. Genomics 282, 503–516. doi: 10.1007/s00438-009-0481-3 19760256

[B31] Jin Z. (2011). Amaryllidaceae and sceletium alkaloids. Nat. Prod. Rep. 28, 1126–1142. doi: 10.1039/c0np00073f 21472174

[B32] Kanehisa M. Furumichi M. Tanabe M. Sato Y. Morishima K. (2017). KEGG: new perspectives on genomes, pathways, diseases and drugs. Nucleic Acids Res. 45, D353–D361. doi: 10.1093/nar/gkw1092 27899662 PMC5210567

[B33] Kim J. Lee J. Choi J. P. Park I. Yang K. Kim M. K.. (2014). Functional innovations of three chronological mesohexaploid *Brassica rapa* genomes. BMC Genomics 15, 606. doi: 10.1186/1471-2164-15-606 25033750 PMC4117954

[B34] Koini M. A. Alvey L. Allen T. Tilley C. A. Harberd N. P. Whitelam G. C.. (2009). High temperature-mediated adaptations in plant architecture require the bHLH transcription factor PIF4. Curr. Biol. 19, 408–413. doi: 10.1016/j.cub.2009.01.046 19249207

[B35] Komatsu M. Maekawa M. Shimamoto K. Kyozuka J. (2001). The LAX1 and FRIZZY PANICLE 2 genes determine the inflorescence architecture of rice by controlling rachis-branch and spikelet development. Dev. Biol. 231, 364–373. doi: 10.1006/dbio.2000.9988 11237465

[B36] Kumar S. Stecher G. Tamura K. (2016a). MEGA7: molecular evolutionary genetics analysis version 7.0 for bigger datasets. Mol. Biol. Evol. 33, 1870–1874. doi: 10.1093/molbev/msw054 27004904 PMC8210823

[B37] Kumar I. Swaminathan K. Hudson K. Hudson M. E. (2016b). Evolutionary divergence of phytochrome protein function in *Zea mays* PIF3 signaling. J. Exp. Bot. 67, 4231–4240. doi: 10.1093/jxb/erw217 27262126 PMC5301934

[B38] Letunic I. Khedkar S. Bork P. (2021). SMART: recent updates, new developments and status in 2020. Nucleic Acids Res. 49, D458–D460. doi: 10.1093/nar/gkaa937 33104802 PMC7778883

[B39] Li S. (2014). Transcriptional control of flavonoid biosynthesis: fine-tuning of the MYB-bHLH-WD40 (MBW) complex. Plant Signal Behav. 9, e27522. doi: 10.4161/psb.27522 24393776 PMC4091223

[B40] Li C. Cai X. Shen Q. Chen X. Xu M. Ye T.. (2022). Genome-wide analysis of basic helix-loop-helix genes in *Dendrobium catenatum* and functional characterization of DcMYC2 in jasmonate-mediated immunity to *Sclerotium delphinii* . Front. Plant Sci. 13. doi: 10.3389/fpls PMC9378844 35982703

[B41] Li X. Duan X. Jiang H. Sun Y. Tang Y. Yuan Z.. (2006). Genome-wide analysis of basic helix-loop-helix transcription factor family in rice and *Arabidopsis* . Plant Physiol. 141, 1167–1184. doi: 10.1104/pp.106.080580 16896230 PMC1533929

[B42] Li H. Gao W. Xue C. Zhang Y. Liu Z. Zhang Y.. (2019b). Genome-wide analysis of the bHLH gene family in Chinese jujube (*Ziziphus jujuba mill.*) and wild jujube. BMC Genomics 20, 568. doi: 10.1186/s12864-019-5936-2 31291886 PMC6617894

[B43] Li W. Qiao C. Pang J. Zhang G. Luo Y. (2019a). The versatile O-methyltransferase LrOMT catalyzes multiple O-methylation reactions in amaryllidaceae alkaloids biosynthesis. Int. J. Biol. Macromol. 141, 680–692. doi: 10.1016/j.ijbiomac.2019.09.011 31494163

[B44] Liu Y. Li X. Li K. Liu H. Lin C. (2013). Multiple bHLH proteins form heterodimers to mediate CRY2-dependent regulation of flowering-time in arabidopsis. PloS Genet. 9, e1003861. doi: 10.1371/journal.pgen.1003861 24130508 PMC3794922

[B45] Liu X. Pan T. Liang W. Gao L. Wang X. Li H.. (2016). Overexpression of an orchid (*Dendrobium nobile*) SOC1/TM3-like ortholog, DnAGL19, in *Arabidopsis* regulates HOS1-FT expression. Front. Plant Sci. 7. doi: 10.3389/fpls.2016.00099 PMC4746357 26904066

[B46] Liu R. Song J. Liu S. Chen C. Zhang S. Wang J.. (2021). Genome-wide identification of the *Capsicum* bHLH transcription factor family: discovery of a candidate regulator involved in the regulation of species-specific bioactive metabolites. BMC Plant Biol. 21, 262. doi: 10.1186/s12870-021-03004-7 34098881 PMC8183072

[B47] Li J. Wang T. Han J. Ren Z. (2020b). Genome-wide identification and characterization of cucumber bHLH family genes and the functional characterization of CsbHLH041 in NaCl and ABA tolerance in *Arabidopsis* and cucumber. BMC Plant Biol. 20, 272. doi: 10.1186/s12870-020-02440-1 32527214 PMC7291561

[B48] Li Q. Xu J. Yang L. Zhou X. Cai Y. Zhang Y. (2020a). Transcriptome analysis of different tissues reveals key genes associated with galanthamine biosynthesis in *Lycoris longituba* . Front. Plant Sci. 11. doi: 10.3389/fpls.2020.519752 PMC7525064 33042169

[B49] Li W. Yang Y. Qiao C. Zhang G. Luo Y. (2018). Functional characterization of phenylalanine ammonia-lyase- and cinnamate 4-hydroxylase-encoding genes from lycoris radiata, a galanthamine-producing plant. Int. J. Biol. Macromol. 117, 1264–1279. doi: 10.1016/j.ijbiomac.2018.06.046 29894786

[B50] Lorenzo O. Chico J. M. Sánchez-Serrano J. J. Solano R. (2004). JASMONATE-INSENSITIVE1 encodes a MYC transcription factor essential to discriminate between different jasmonate-regulated defense responses in *Arabidopsis* . Plant Cell 16, 1938–1950. doi: 10.1105/tpc.022319 15208388 PMC514172

[B51] Lu R. Zhang J. Liu D. Wei Y. L. Wang Y. Li X. B. (2018). Characterization of bHLH/HLH genes that are involved in brassinosteroid (BR) signaling in fiber development of cotton (*Gossypium hirsutum*). BMC Plant Biol. 18, 304. doi: 10.1186/s12870-018-1523-y 30482177 PMC6258498

[B52] Menand B. Yi K. Jouannic S. Hoffmann L. Ryan E. Linstead P.. (2007). An ancient mechanism controls the development of cells with a rooting function in land plants. Science 316, 1477–1480. doi: 10.1126/science.1142618 17556585

[B53] Mistry J. Chuguransky S. Williams L. Qureshi M. Salazar G. A. Sonnhammer E. L. L.. (2021). Pfam: the protein families database in 2021. Nucleic Acids Res. 49, D412–D419. doi: 10.1093/nar/gkaa913 33125078 PMC7779014

[B54] Morohashi K. Zhao M. Yang M. Read B. Lloyd A. Lamb R.. (2007). Participation of the *Arabidopsis* bHLH factor GL3 in trichome initiation regulatory events. Plant Physiol. 145, 736–746. doi: 10.1104/pp.107.104521 17885086 PMC2048771

[B55] Murre C. McCaw P. S. Baltimore D. (1989). A new DNA binding and dimerization motif in immunoglobulin enhancer binding, daughterless, MyoD, and myc proteins. Cell 56, 777–783. doi: 10.1016/0092-8674(89)90682-X 2493990

[B56] Niu X. Guan Y. Chen S. Li H. (2017). Genome-wide analysis of basic helix-loop-helix (bHLH) transcription factors in *Brachypodium distachyon* . BMC Genomics 18, 619. doi: 10.1186/s12864-017-4044-4 28810832 PMC5558667

[B57] Park C. H. Yeo H. J. Park Y. E. Baek S. A. Kim J. K. Park S. U. (2019). Transcriptome analysis and metabolic profiling of *Lycoris radiata* . Biology 8, 63. doi: 10.3390/biology8030063 31470601 PMC6784096

[B58] Pillitteri L. J. Sloan D. B. Bogenschutz N. L. Torii K. U. (2007). Termination of asymmetric cell division and differentiation of stomata. Nature 445, 501–505. doi: 10.1038/nature05467 17183267

[B59] Pires N. Dolan L. (2010). Origin and diversification of basic-helix-loop-helix proteins in plants. Mol. Biol. Evol. 27, 862–874. doi: 10.1093/molbev/msp288 19942615 PMC2839125

[B60] Qi X. Torii K. U. (2018). Hormonal and environmental signals guiding stomatal development. BMC Biol. 16, 21. doi: 10.1186/s12915-018-0488-5 29463247 PMC5819259

[B61] Ramsay N. A. Glover B. J. (2005). MYB-bHLH-WD40 protein complex and the evolution of cellular diversity. Trends Plant Sci. 10, 63–70. doi: 10.1016/j.tplants.2004.12.011 15708343

[B62] Ren Z. Xu Y. Lvy X. Zhang D. Gao C. Lin Y.. (2021). Early sucrose degradation and the dominant sucrose cleavage pattern influence *Lycoris sprengeri* bulblet regeneration *in vitro* . Int. J. Mol. Sci. 22, 11890. doi: 10.3390/ijms222111890 34769318 PMC8585118

[B63] Ren Z. M. Zhang D. Jiao C. Li D. Q. Wu Y. Wang X. Y.. (2022). Comparative transcriptome and metabolome analyses identified the mode of sucrose degradation as a metabolic marker for early vegetative propagation in bulbs of *Lycoris* . Plant J. 112, 115–134. doi: 10.1111/tpj.15935 35942603 PMC9826282

[B64] Sakamoto W. Ohmori T. Kageyama K. Miyazaki C. Saito A. Murata M.. (2001). The purple leaf (Pl) locus of rice: the pl (w) allele has a complex organization and includes two genes encoding basic helix-loop-helix proteins involved in anthocyanin biosynthesis. Plant Cell Physiol. 42, 982–991. doi: 10.1093/pcp/pce128 11577193

[B65] Schmittgen T. D. Livak K. J. (2008). Analyzing real-time PCR data by the comparative C(T) method. Nat. Protoc. 3, 1101–1108. doi: 10.1038/nprot.2008.73 18546601

[B66] Schuster C. Gaillochet C. Lohmann J. U. (2015). *Arabidopsis HECATE* genes function in phytohormone control during gynoecium development. Development 142, 3343–3350. doi: 10.1242/dev.120444 26293302 PMC4631749

[B67] Seo J. S. Joo J. Kim M. J. Kim Y. K. Nahm B. H. Song S. I.. (2011). OsbHLH148, a basic helix-loop-helix protein, interacts with OsJAZ proteins in a jasmonate signaling pathway leading to drought tolerance in rice. Plant J. 65, 907–921. doi: 10.1111/j.1365-313X.2010.04477.x 21332845

[B68] Shen C. Y. Xu X. L. Yang L. J. Jiang J. G. (2019). Identification of narciclasine from *Lycoris radiata* (L’Her.) herb. and its inhibitory effect on LPS-induced inflammatory responses in macrophages. Food Chem. Toxicol. 125, 605–613. doi: 10.1016/j.fct.2019.02.003 30738987

[B69] Shi S. D. Qiu Y. X. Wu L. Fu C. X. (2006). Interspecific relationships of *Lycoris* (amaryllidaceae) inferred from inter-simple sequence repeat data. Sci. Hortic. 110, 285–291. doi: 10.1016/j.scienta.2006.07.011

[B70] Shi Q. Zhang H. Song X. Ye J. Liang R. Li G. (2018). Functional characterization of the maize phytochrome-interacting factors PIF4 and PIF5. Front. Plant Sci. 8. doi: 10.3389/fpls.2017.02273 PMC5778267 29403515

[B71] Smolen G. A. Pawlowski L. Wilensky S. E. Bender J. (2002). Dominant alleles of the basic helix-loop-helix transcription factor ATR2 activate stress-responsive genes in *Arabidopsis* . Genetics 161, 1235–1246. doi: 10.1093/genetics/161.3.1235 12136026 PMC1462177

[B72] Song X. M. Huang Z. N. Duan W. K. Ren J. Liu T. K. Li Y.. (2014). Genome-wide analysis of the bHLH transcription factor family in Chinese cabbage (Brassica rapa ssp. pekinensis). Mol. Genet. Genomics 289, 77–91. doi: 10.1007/s00438-013-0791-3 24241166

[B73] Sorensen A. M. Kröber S. Unte U. S. Huijser P. Dekker K. Saedler H. (2003). The *Arabidopsis aborted microspores* (*AMS*) gene encodes a MYC class transcription factor. Plant J. 33, 413–423. doi: 10.1046/j.1365-313x.2003.01644.x 12535353

[B74] Sun H. Fan H. J. Ling H. Q. (2015). Genome-wide identification and characterization of the bHLH gene family in tomato. BMC Genomics 16, 9. doi: 10.1186/s12864-014-1209-2 25612924 PMC4312455

[B75] Sun X. Wang Y. Sui N. (2018). Transcriptional regulation of bHLH during plant response to stress. Biochem. Biophys. Res. Commun. 503, 397–401. doi: 10.1016/j.bbrc.2018.07.123 30057319

[B76] Szklarczyk D. Gable A. L. Nastou K. C. Lyon D. Kirsch R. Pyysalo S.. (2021). The STRING database in 2021: customizable protein-protein networks, and functional characterization of user-uploaded gene/measurement sets. Nucleic Acids Res. 49, 10800. doi: 10.1093/nar/gkab835 34530444 PMC8501959

[B77] Toledo-Ortiz G. Huq E. Quail P. H. (2003). The *Arabidopsis* basic/helix-loop-helix transcription factor family. Plant Cell. 15, 1749–1770. doi: 10.1105/tpc.013839 12897250 PMC167167

[B78] Valea I. Motegi A. Kawamura N. Kawamoto K. Miyao A. Ozawa R.. (2021). The rice wound-inducible transcription factor *RERJ1* sharing same signal transduction pathway with OsMYC2 is necessary for defense response to herbivory and bacterial blight. Plant Mol. Biol. 109, 651–666. doi: 10.1007/s11103-021-01186-0 34476681

[B79] Varaud E. Brioudes F. Szécsi J. Leroux J. Brown S. Perrot-Rechenmann C.. (2011). AUXIN RESPONSE FACTOR8 regulates *Arabidopsis* petal growth by interacting with the bHLH transcription factor BIGPETALp. Plant Cell 23, 973–983. doi: 10.1105/tpc.110.081653 21421811 PMC3082276

[B80] Verma D. Jalmi S. K. Bhagat P. K. Verma N. Sinha A. K. (2020). A bHLH transcription factor, MYC2, imparts salt intolerance by regulating proline biosynthesis in *Arabidopsis* . FEBS J. 287, 2560–2576. doi: 10.1111/febs.15157 31782895

[B81] Wang R. Han X. Xu S. Xia B. Jiang Y. Xue Y.. (2019b). Cloning and characterization of a tyrosine decarboxylase involved in the biosynthesis of galanthamine in *Lycoris aurea* . PeerJ 7, e6729. doi: 10.7717/peerj.6729 31024762 PMC6474336

[B82] Wang Y. Liu A. (2020). Genomic characterization and expression analysis of basic Helix-Loop-Helix (bHLH) family genes in traditional chinese herb dendrobium officinale. Plants (Basel). 9, 1044. doi: 10.3390/plants9081044 32824436 PMC7463459

[B83] Wang N. Shu X. Zhang F. Zhuang W. Wang T. Wang Z. (2021). Comparative transcriptome analysis identifies key regulatory genes involved in anthocyanin metabolism during flower development in *Lycoris radiata* . Front. Plant Sci. 12. doi: 10.3389/fpls.2021.761862 PMC8715008 34975946

[B84] Wang P. Su L. Gao H. Jiang X. Wu X. Li Y.. (2018). Genome-wide characterization of *bHLH* genes in grape and analysis of their potential relevance to abiotic stress tolerance and secondary metabolite biosynthesis. Front. Plant Sci. 9. doi: 10.3389/fpls.2018.00064 PMC5799661 29449854

[B85] Wang L. Xiang L. Hong J. Xie Z. Li B. (2019a). Genome-wide analysis of bHLH transcription factor family reveals their involvement in biotic and abiotic stress responses in wheat (*Triticum aestivum* l.). 3 Biotech. 9, 236. doi: 10.1007/s13205-019-1742-4 PMC6536565 31139551

[B86] Wang R. Xu S. Wang N. Xia B. Jiang Y. Wang R. (2017). Transcriptome analysis of secondary metabolism pathway, transcription factors, and transporters in response to methyl jasmonate in *Lycoris aurea* . Front. Plant Sci. 1971 7. doi: 10.3389/fpls.2016.01971 28111578 PMC5217099

[B87] Wang P. Yu S. Han X. Xu J. He Q. Xu S.. (2020). Identification, molecular characterization and expression of *JAZ* genes in *Lycoris aurea* . PloS One 15, e0230177. doi: 10.1371/journal.pone.0230177 32182273 PMC7077819

[B88] Xie X. B. Li S. Zhang R. F. Zhao J. Chen Y. C. Zhao Q.. (2012). The bHLH transcription factor MdbHLH3 promotes anthocyanin accumulation and fruit colouration in response to low temperature in apples. Plant Cell Environ. 35, 1884–1897. doi: 10.1111/j.1365-3040.2012.02523.x 22519753

[B89] Yang Y. F. Zhang K. K. Yang L. Y. Lv X. Wu Y. Liu H. W.. (2018). Identification and characterization of MYC transcription factors in taxus sp. Gene 675, 1–8. doi: 10.1016/j.gene.2018.06.065 29935357

[B90] Yue Y. Liu J. Shi T. Chen M. Li Y. Du J.. (2019). Integrating transcriptomic and GC-MS metabolomic analysis to characterize color and aroma formation during tepal development in *Lycoris longituba* . Plants 8, 53. doi: 10.3390/plants8030053 30823447 PMC6473938

[B91] Zander M. Lewsey M. G. Clark N. M. Yin L. Bartlett A. Guzmán J. P. S.. (2020). Integrated multi-omics framework of the plant response to jasmonic acid. Nat. Plants 6, 290–302. doi: 10.1038/s41477-020-0605-7 32170290 PMC7094030

[B92] Zhang H. B. Bokowiec M. T. Rushton P. J. Han S. C. Timko M. P. (2012). Tobacco transcription factors NtMYC2a and NtMYC2b form nuclear complexes with the NtJAZ1 repressor and regulate multiple jasmonate-inducible steps in nicotine biosynthesis. Mol. Plant 5, 73–84. doi: 10.1093/mp/ssr056 21746701

[B93] Zhang T. T. Lv W. Zhang H. S. Ma L. Li P. H. Ge L.. (2018a). Genome-wide analysis of the basic helix-loop-helix (bHLH) transcription factor family in maize. BMC Plant Biol. 18, 235. doi: 10.1186/s12870-018-1441-z 30326829 PMC6192367

[B94] Zhang T. Mo J. Zhou K. Chang Y. Liu Z. (2018b). Overexpression of *Brassica campestris BcICE1* gene increases abiotic stress tolerance in tobacco. Plant Physiol. Biochem. 132, 515–523. doi: 10.1016/j.plaphy.2018.09.039 30312954

[B95] Zhao H. Wang X. Zhu D. Cui S. Li X. Cao Y.. (2012). A single amino acid substitution in IIIf subfamily of basic helix-loop-helix transcription factor AtMYC1 leads to trichome and root hair patterning defects by abolishing its interaction with partner proteins in *Arabidopsis* . J. Biol. Chem. 287, 14109–14121. doi: 10.1074/jbc.M111.280735 22334670 PMC3340177

