# Supplementary figures and images for: Transcriptome-wide characterization of bHLH transcription factor genes in Lycoris radiata and functional analysis of their response to MeJA
Source: Front Plant Sci. 2023 Jan 10;13:975530. doi: 10.3389/fpls.2022.975530 (PMC9872026; doi:10.3389/fpls.2022.975530)

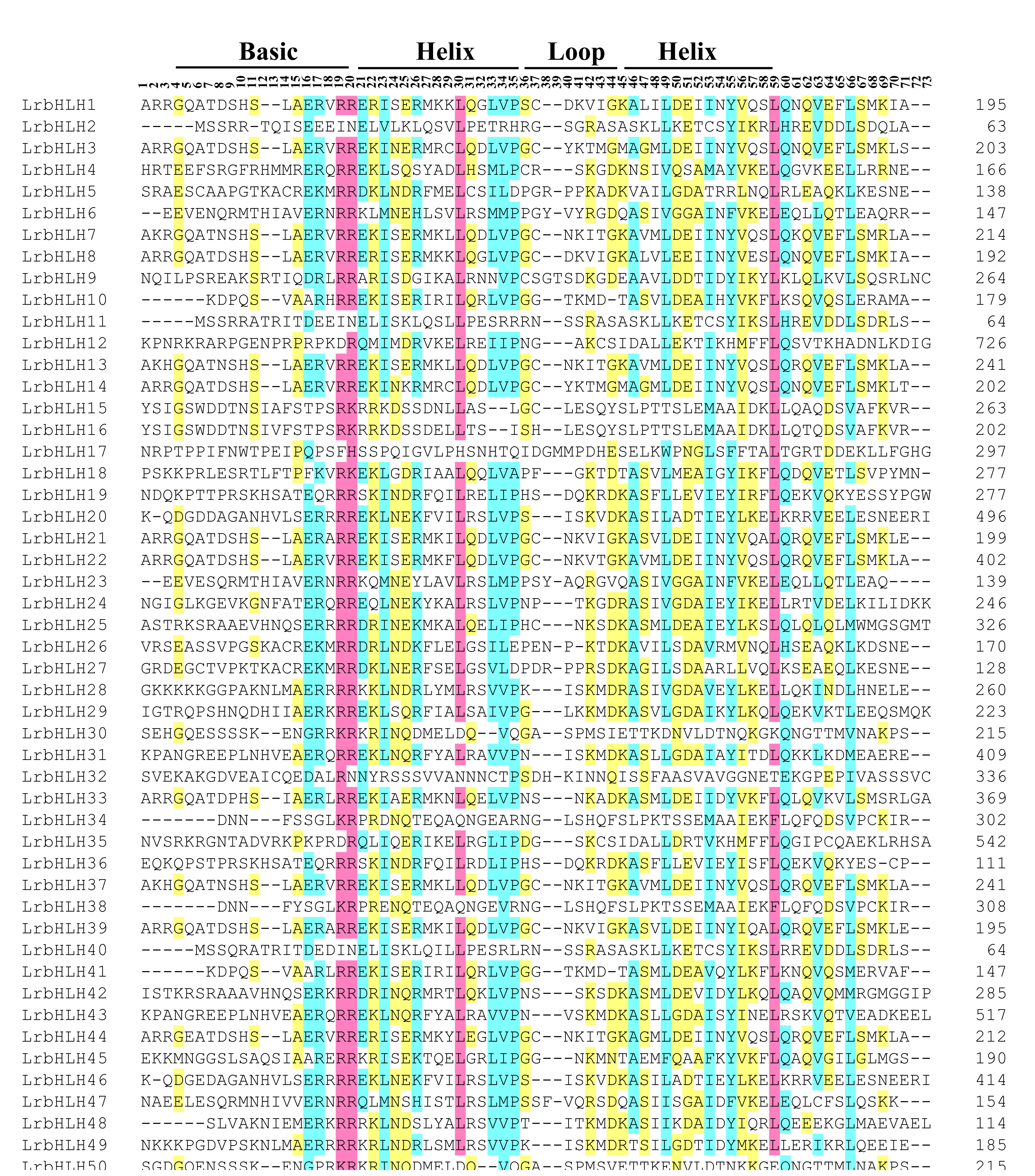

Supplement: Supplementary Figure 1 — Multiple sequence alignment of the bHLH conserved domain in L.radiata bHLH proteins. The yellow boxes indicate 30% identity of amino acids, the blue boxes indicate 50% identity of amino acids, and the red boxes indicate 75% identity of amino acids. [file Image_1.tif]

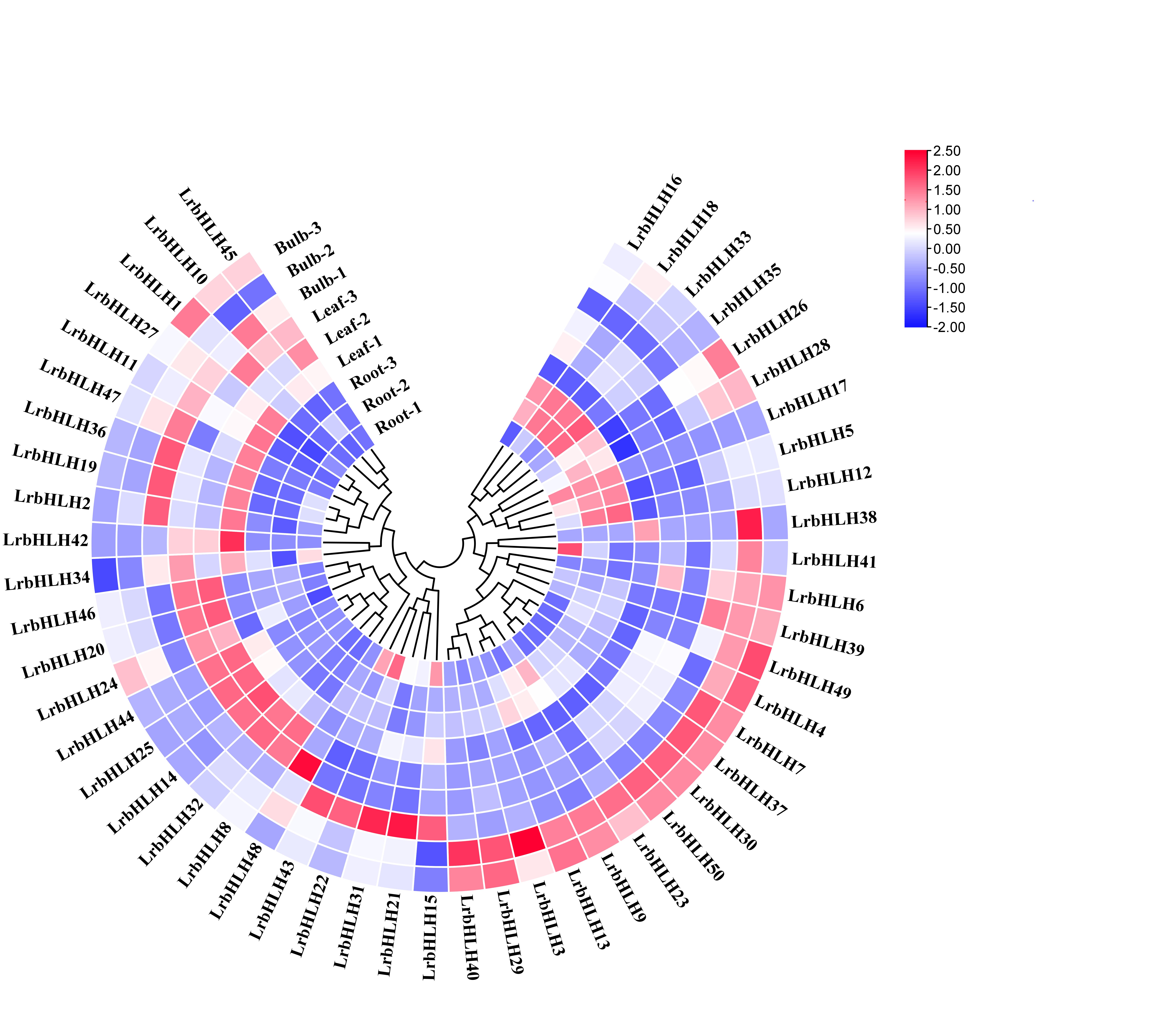

Supplement: Supplementary Figure 2 — Expression profile heatmap with hierarchal clustering of LrbHLHs in different tissues of L. longituba. Red and blue represent high and low relative transcript abundance, respectively. [file Image_2.jpeg]

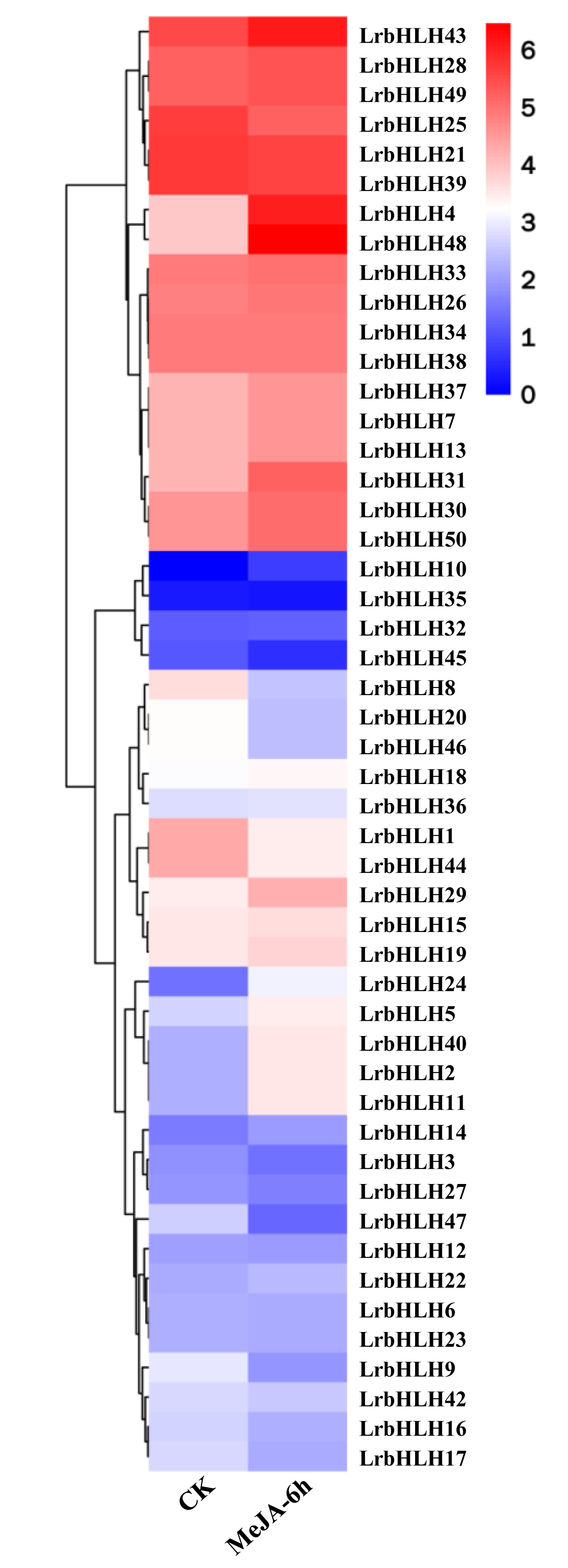

Supplement: Supplementary Figure 3 — Heatmap of LrbHLH gene expression profiles with MeJA treatment. Red and blue represent high and low relative transcript abundance, respectively. [file Image_3.tif]

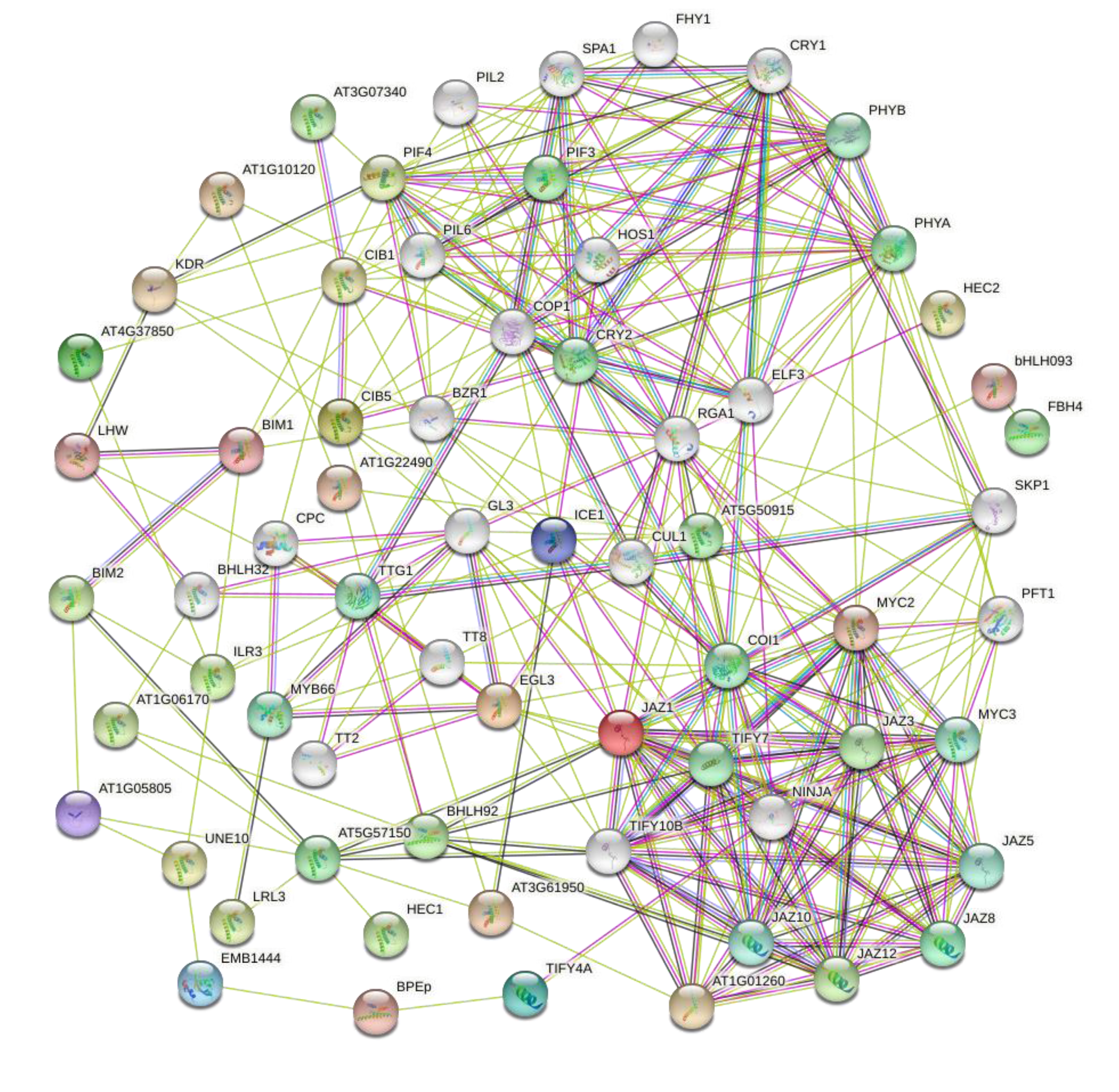

Supplement: Supplementary Figure 4 — The predicted network of protein-protein interactions between LrbHLHs by STRING database. [file Image_4.tif]
